# Impact of the SARS-CoV-2 nucleocapsid 203K/204R mutations on the inflammatory immune response in COVID-19 severity

**DOI:** 10.1186/s13073-023-01208-0

**Published:** 2023-07-21

**Authors:** Muhammad Shuaib, Sabir Adroub, Tobias Mourier, Sara Mfarrej, Huoming Zhang, Luke Esau, Afrah Alsomali, Fadwa S Alofi, Adeel Nazir Ahmad, Abbas Shamsan, Asim Khogeer, Anwar M. Hashem, Naif A. M. Almontashiri, Sharif Hala, Arnab Pain

**Affiliations:** 1 Pathogen Genomics Laboratory, Bioscience Program, Biological and Environmental Science and Engineering (BESE), King Abdullah University of Science and Technology (KAUST), Thuwal, 23955-6900 Saudi Arabia; 2 Bioscience Core Laboratory, King Abdullah University of Science and Technology (KAUST), Thuwal, 23955-6900 Saudi Arabia; 3 Infectious Diseases Department, King Abdullah Medical Complex, Jeddah, MOH Saudi Arabia; 4 Infectious Diseases Department, King Fahad Hospital, Madinah, MOH Saudi Arabia; 5 KAUST Health - Fakeeh Care, King Abdullah University of Science and Technology, Thuwal, Saudi Arabia; 6 Dr. Suliman Al-Habib Medical Group, Riyadh, Saudi Arabia; 7 Plan and Research Department, General Directorate of Health Affairs Makkah Region, Makkah, MOH Saudi Arabia; 8 Vaccines and Immunotherapy Unit, King Fahd Medical Research Center, King Abdulaziz University, Jeddah, Saudi Arabia; 9 Department of Clinical Microbiology and Immunology, Faculty of Medicine, King Abdulaziz University, Jeddah, Saudi Arabia; 10 College of Applied Medical Sciences, Taibah University, Madinah, Saudi Arabia; 11 Center for Genetics and Inherited Diseases, Taibah University, Almadinah Almunwarah, Saudi Arabia; 12 Infectious Disease Research Department, King Abdullah International Medical Research Centre, Ministry of National Guard Health Affairs, Jeddah, Saudi Arabia; 13 King Saud Bin Abdulaziz University for Health Sciences, Ministry of National Guard Health Affairs, Jeddah, Saudi Arabia; 14 International Institute for Zoonosis Control, Global Institution for Collaborative Research and Education (GI-CoRE), Hokkaido University, Sapporo, 001-0020 Japan

**Keywords:** COVID-19, SARS-CoV-2, Variant of concern, Nucleocapsid (N) R203K/G204R (KR) mutations, Virus-like particle (VLP), Transcriptomics, Proteomics, Host immune response, Interferon-stimulated genes (ISGs), Cytokine storm

## Abstract

**Background:**

The excessive inflammatory responses provoked by SARS-CoV-2 infection are critical factors affecting the severity and mortality of COVID-19. Previous work found that two adjacent co-occurring mutations R203K and G204R (KR) on the nucleocapsid (N) protein correlate with increased disease severity in COVID-19 patients. However, links with the host immune response remain unclear.

**Methods:**

Here, we grouped nasopharyngeal swab samples of COVID-19 patients into two cohorts based on the presence and absence of SARS-CoV-2 nucleocapsid KR mutations. We performed nasopharyngeal transcriptome analysis of age, gender, and ethnicity-matched COVID-19 patients infected with either SARS-CoV-2 with KR mutations in the N protein (KR patients *n* = 39) or with the wild-type N protein (RG patients *n* = 39) and compared to healthy controls (*n* = 34). The impact of KR mutation on immune response was further characterized experimentally by transcriptomic and proteomic profiling of virus-like-particle (VLP) incubated cells.

**Results:**

We observed markedly elevated expression of proinflammatory cytokines, chemokines, and interferon-stimulated (ISGs) genes in the KR patients compared to RG patients. Using nasopharyngeal transcriptome data, we found significantly higher levels of neutrophils and neutrophil-to-lymphocyte (NLR) ratio in KR patients than in the RG patients. Furthermore, transcriptomic and proteomic profiling of VLP incubated cells confirmed a similar hyper-inflammatory response mediated by the KR variant.

**Conclusions:**

Our data demonstrate an unforeseen connection between nucleocapsid KR mutations and augmented inflammatory immune response in severe COVID-19 patients. These findings provide insights into how mutations in SARS-CoV-2 modulate host immune output and pathogenesis and may contribute to more efficient therapeutics and vaccine development.

**Supplementary Information:**

The online version contains supplementary material available at 10.1186/s13073-023-01208-0.

## Background

The COVID-19 pandemic is arguably the most important global threat to humankind the world has seen in the twenty-first century. Since the emergence of the severe acute respiratory syndrome coronavirus 2 (SARS-CoV-2) in December 2019, COVID-19 has caused more than 604 million confirmed cases, with more than 6.64 million deaths to date [1]. The SARS-CoV-2 RNA genome is continuously mutating, and some mutations may enable the resulting novel variants to better adapt for infection and transmission [2]. Current research predominantly focuses on spike mutations as they may impact transmission or evade the current vaccine’s immunity [3]. While spike protein changes are critical in transmission or immune evasion [4], the emerging viral variants also contain mutations outside the spike protein, which may affect COVID-19 pathogenesis by modulating virus-host interaction and host antiviral response. For example, SARS-CoV-2 accessory proteins [5–9] and non-structural protein Nsp1 [10] have been shown to affect antiviral host responses. The ORF8 accessory protein was recently reported to induce a hyper-inflammatory cytokine storm by binding to human dendritic cells [11]. Besides spike (S), other structural proteins such as envelope (E), membrane (M), and nucleocapsid (N) contain mutations in the newly emerging virus variants [12].

We recently found that two consecutive amino acid mutations R203K and G204R (for simplicity the R203K/G204R mutant is called KR and the wild-type nucleocapsid is termed RG) in the nucleocapsid (N) protein, are linked with increased viral load and severity in a diverse population of COVID-19 patients [13]. The exact mechanism of COVID-19 disease severity remains unclear. However, studies have indicated that dysregulated host immune response and production of inflammatory cytokines, the so-called cytokine storms, is critical for disease severity and death in COVID-19 patients [14, 15]. Indeed, enhanced expression of interleukins, tumor necrosis factors, cytokines, and various chemokines was reported in patients with severe COVID-19 [15–17]. N protein from SARS-CoV-2 and SARS-CoV has been shown to elicit host immune response and contain high immunogenic properties [18, 19]. The KR mutation in N protein is also present in various SARS-CoV-2 variants of concern (VOC) such as Alpha, Gamma, Lambda, and Omicron in various proportions [20]. It was recently reported to be linked with an enhanced subgenomic RNA expression [21]. Another study using the cell culture and animal model system showed that KR mutations enhance infectivity, fitness, and virulence [22]. SARS-CoV-2 virus-like particle (VLP) approach has also revealed enhanced infectivity of KR mutation in the presence and absence of omicron background mutations [23, 24]. The identified association of KR mutation with increased disease severity suggests a potential link with host immune response, which is so far unexplored.

In this work, by employing a combination of omics approaches using patient samples and cell culture models, we provide evidence for an unexpected connection of KR mutation with augmented host immune response in COVID-19 patients. We performed a comparative transcriptomic analysis of COVID-19 patients infected with KR-mutant SARS-CoV-2 (KR-patients *n* = 39) or wild-type SARS-CoV-2 (RG-patients *n* = 39) compared to healthy controls (Healthy *n* = 34). Our data demonstrate noticeable disparities in host transcriptional responses, especially the immune response genes, between the KR-patients and RG-patients. The KR-patients display high-level overexpression of immune response than RG-patients. The impact of KR mutation on immune response was further characterized experimentally in cell lines using the VLP approach. By transcriptomic and proteomic profiling of VLP incubated cells, we identified and validated our observed association of enhanced immune response orchestrated by the KR variant. These findings reveal an unprecedented link between the nucleocapsid KR mutations in SARS-CoV-2 and a clinically significant hyper-immune phenotype of COVID-19, which, on the one hand, may explain disease severity during the early wave of the pandemic and, on the other hand, may aid in an effective therapeutic and broad vaccine development.

## Methods

### Cohorts

As described previously [13], nasopharyngeal swab samples were collected (during March to August 2020) from 78 COVID-19 patients and 34 healthy controls in 1 ml of TRIzol (Ambion, USA) in Saudi Arabia. The anonymized samples were obtained from 7 hospitals and one quarantine hotel in Makkah region. Two COVID-19 patient cohorts based on the nucleocapsid (N) protein mutation profiling (R203K/G204R), reported previously [13], were used in this study. The demographic details of the patient cohorts are provided in Additional file 2: Table S1 and Additional file 1: Fig. S1. COVID-19 samples from patients infected with SARS-CoV-2 having two consecutive amino acid mutations in the N protein (203 K/204R) called KR patients (*n* = 39). The patients infected with SARS-CoV-2 having wild-type N protein sequence (R203/G204) called RG patients (*n* = 39). The healthy control samples (*n* = 34) were collected from unidentified individuals without any known respiratory symptoms.

RNA from nasopharyngeal swab samples was extracted using the optimized [13] Direct-Zol RNA Miniprep kit (Zymo Research, USA) following the manufacturer’s instructions. All samples were analyzed by real-time PCR for SARS-CoV-2 detection using primers for viral nucleocapsid gene (N1 and N2) and primer for human RNase P gene (CDC 2019 nCoV Real-Time RT-PCR Diagnostic Panel).

### Metatranscriptome sequencing

RNA-seq libraries were prepared using TruSeq Stranded Total RNA with Ribo-Zero Plus (Illumina, USA) according to the manufacturer’s instructions. Libraries were pooled and processed for metatranscriptome sequencing using the Illumina Novaseq 6000 platform on the SP4 (2 × 150 bp) flow cell (Illumina, USA).

### Data processing and differential analysis

The raw sequencing reads were processed for quality control analysis. The adaptor trimming and low-quality reads removal from fastq files was performed by using trimmomatic [25]. The clean reads were mapped to human genome (hg19) [26, 27] annotated ENSEMBL transcripts using kallisto [28] with default parameters. The generated mapped bam files were sorted using Samtools [29]. The gene counts were normalized using the log 2 normalization method (90). Differential gene expression analysis was performed using the R package EdgeR integrated in the NetworkAnalyst [30]. Functional enrichment and pathway enrichment analyses of differentially expressed genes were carried out using NetworkAnalyst [30].

For distance clustering, the raw read counts were TMM normalized using the R package edgeR [31], and distances between samples based on a selection of ISGs and cytokine genes were calculated using the R function “dist” with the “canberra” method. From these distances, samples were clustered using the R function hclust with the “complete” method. The resulting tree was converted to newick format using the 'as.phylo' function from the R package “ape” (v5.6–2) [32]. All analyses were carried out in Rstudio (RStudio Team 2020; R Core Team 2020). Finally, the tree was formatted in FigTree (v1.4.2).

### Prediction of immune cells from transcriptome data

To predict and estimate the proportion of immune cell types, the raw nasopharyngeal transcriptome count data were processed by using the CIBERSORT algorithm (version v1.06) [33]. The analysis was performed using the original gene signature file LM22 of CIBERSORT and 100 permutations. The proportion of predicted immune cell types was represented in bar graph.

### Custom virus-like particles (VLPs)

We obtained four customized strains of hybrid alphavirus-SARS-CoV-2 (Ha-CoV-2, Luc) virus-like particles (VLP) [34] from Virongy Biosciences Inc. These include (1) wild-type VLP (VLP-WT) containing reference sequences of all four structural proteins (S, M, N, and E), (2) KR-mutant (KR-VLP) with two mutations R203K/G204R in the nucleocapsid protein, (3) D614G-KR VLP containing both R203K/G204R and spike D614G mutations, and (4) D614G VLP contains only spike D614G mutation.

### Cell culture, transfection, and VLP incubation

HEK293T (ACE2 + TMPRSS2) (Catalog# RCSNAK-01) and HEK293T (ATCC; CRL-3216).cell lines were maintained in DMEM supplemented with 10% fetal bovine serum (FBS; Cell-Box) and 1% penicillin–streptomycin (Thermo Fisher Scientific). Cells were grown according to standard protocols at 37°C and 5% CO2. Transfection of one million cells per well in 6-well plate with 2 × Strep-tagged N plasmid (2ug/transfection) was performed using lipofectamine-2000 according standard protocol.

VLP particles (WT-VLP, KR-VLP, D614G/KR-VLP, and D614G-VLP) were used to incubate HEK293T (ACE2 + TMPRSS2). Cells were first plated in 6-well plate (0.3 × 10^6^ cells per well) and culture overnight. The next day, media was removed from the cells and incubated with VLP by adding 100 ul VLP particles in 400 µl of growth media. The cells were incubated in VLP containing media overnight and then collected for downstream processes.

### Luciferase assay

Following the incubation with VLP media overnight, cells were lysed in 200 μl of 1 × cell lysis buffer (Luciferase Assay, Promega) and used 50 μl for luciferase assays on GloMax Discover Microplate Reader (Promega) according to the manufacturer’s instructions.

### RNA-sequencing (RNA-seq) from transfected and VLP incubated cells

Cells were lysed in 1 ml of TRIzol (Ambion, USA) and total RNA was extracted as mentioned above. Libraries were prepared using TruSeq Stranded Total RNA with Ribo-Zero Plus (Illumina, USA) and sequenced on Novaseq 6000 platform on the SP4 (2 × 150 bp) flow cell (Illumina, USA). Data processing and differential analysis was performed as described above.

### Protein extraction and digestion

HEK293T cell lines stably expressing ACE2/TMPRSS2 were incubated with different VLPs (WT, KR, D614G-KR, and D614G). Briefly, after incubation (24 h), the cells were collected with 10 mM EDTA in 1 × PBS and washed twice with cold PBS (1 ×) in the presence of protease inhibitor. The cell pellets were stored at − 80°C. The cells were lysed in lysis buffer 50 mM Tris–HCl (pH 7.4), 150 mM NaCl, 1 mM EDTA, 1% NP-40, 1% Na-deoxycholate, 0.1% SDS, supplemented with protease and phosphatase inhibitor cocktails for 30 min while rotating at 4°C and then sonicated and centrifuged at high speed to collect the supernatant. Protein concentration was determined using BCA protein assay kit (Pierce™, Catalog: 23225). The protein extract was purified using methanol/chloroform precipitation and dried under vacuum. The dried pellets were resuspended into the extraction buffer (50 mM triethylammonium bicarbonate and 5% SDS in Water). The protein content was determined using a nanodrop (Thermo Scientific) and then digested using STrap as described [35].

### Mass spectrometery analysis and data processing

Approximately 200 ng of peptide mixture per sample was analyzed using a timsTOF Pro 2 QTOF mass spectrometer coupled with a nanoElute liquid chromatography system (Bruker Daltonik GmbH, Germany).

The sample was injected directly into a RP-C18 Aurora emitter column (75 µm i.d. × 250 mm, 1.6 μm, 120 Å pore size) (Ion Opticks, Australia) using a one-column separation method. An 80-min gradient was established using mobile phase A (0.1% formic acid in H2O) and mobile phase B (0.1% formic acid in Acetonitrile): 2–25% B for 60 min, 25–37% for 10 min, ramping 37% to 95% in 5 min, and maintaining 95% B for 5 min. The column temperature was set at 50°C and the flow rate at 250 nl/min. The sample eluting from the separation column was introduced into the mass spectrometer via a CaptiveSpray nano-electrospray ion source (Bruker Daltonik GmbH) with an electrospray voltage of 1.6 kV. The ion source temperature set to 180 °C and a dry gas of 3 l/min.

The samples were analyzed using diaPASEF scheme [36] consisting of 24 cycles including a total of 48 mass width windows (13 Da (m/z) from m/z 400 to 1000 and TIMS scan range from 0.63 to 1.35 Vs cm − 2 (1/K0). The collisional energy increased linearly from 20.01 eV at 0.6 (1/K0) to 52.00 eV at 1.35 Vs cm − 2 (1/K0). The scan range for MS and MS/MS spectra was set to 100–1700 m/z. TIMS ramping time and accumulation time were set to 100 ms. The diaPASEF data were analyzed by directDIA approach using Spectronaut software (version 15.5.211111, Biognosys, Switzerland) as described [37].

### Cytokines analysis by flow cytometry

Control and VLP (WT and KR) incubated cell lysates were harvested after 24-h post-infection. The level of cytokines was measured using flowcytometry-based cytokine storm multiplex kit (AssayGenie, cytokine storm multiplex panel 9-plex, cat# HUAMCOV05) according to the manufacturer’s instructions. Sample readout was measured by acquiring > 100 events for each bead population in the 585 nm and 695 nm emission channels on an Attune NXT cytometer (ThermoFisher) equipped with 561 nm laser. Data was analyzed with FlowJo v10.1 software (FlowJo).

### Quantification and statistical analysis

RNA-seq and mass spectrometry data were analyzed using specific pipelines with statistical setting described in the methods. The significance of statistical analyses was computed with *t*-test using GraphPad Prism 9. The quantification and statistical details (*p*-value and the value of *n*) of each analysis can be found in the respective figure legends.

## Results

### Transcriptome analysis of COVID-19 patients infected with SARS-CoV-2 variants

**Fig. 1:**
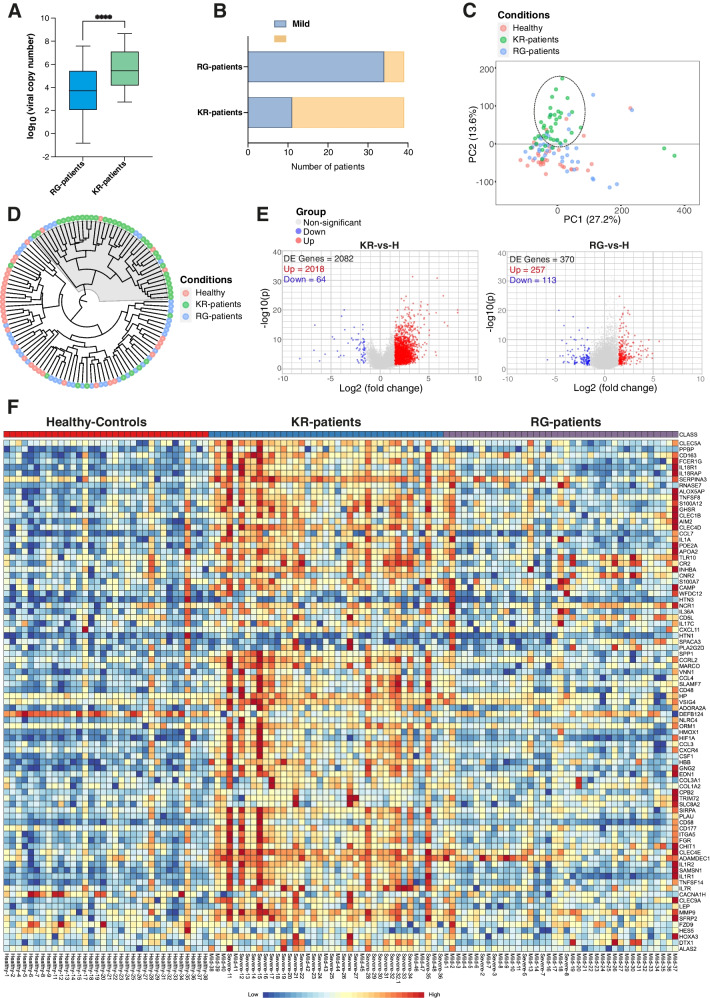
Transcriptome analysis of COVID-19 patients infected with KR-mutant and wild-type SARS-CoV-2.** A** Boxplot showing viral copy numbers (log10 transformed) of RG-patients and KR-patients (two-tailed *t*-test, KR-patients versus RG-patients, *p*-value < 0.0001 (****)). The copy numbers were calculated using standard curve from Ct values (RT-qPCR) of the N1 primer pairs after normalization with RNase P Ct values. Extracted RNA (by Direct-Zol kit, Zymo Research) was subjected to RT-qPCR using one-step TaqPath kit (Applied Biosystems) and nucleocapsid gene (N1) and RNAseP primers. **B** Plot showing the number of mild and severe cases in the RG and KR COVID-19 patient cohorts (severe = ICU/deceased and mild = not admitted to ICU).** C** PCA on transcriptome of nasopharyngeal samples from Healthy control, KR-Patients, and RG-Patients. PCA plot is based on all differentially expressed (DE) genes and autoscaling of data was used. **D** Tree shows hierarchical clustering between samples (Healthy control, KR-Patients, and RG-Patients) based on the expression level of cytokine and interferon stimulated (ISG) genes. The sample names are colored according to the group. The majority of KR-patients samples clustered together as shown in shaded.** E** Volcano plot showing significant (adj *p*-value < 0.05 and log2 fold-change cutoff ≥ 1.5) differentially expressed (DE) genes comparing KR-Patients versus Healthy controls (KR-vs-H) and RG-Patients versus Healthy controls (RG-vs-H) as determined by the method EdgeR. Genes with significant up-regulation are shown in red and down-regulated are shown in blue. All other non-significant genes are shown in gray.** F** Heatmap shows normalized expression of top significantly differentially expressed genes in KR-patient versus Healthy controls (KR-vs-H) (adj *p*-value < 0.05 and log2 fold-change ≥ 1.5). KR-Patients (*n* = 39), RG-Patients (*n* = 39), and Healthy controls (*n* = 34). The sample names represent COVID-19 disease status (severe, mild, and healthy) as in **B**. The heatmap was generated by the visualization module in the ExpressAnalyst

Recently, we highlighted the important link between the KR mutation in the SARS-CoV-2 N protein and disease severity [13]. However, any possible connection of KR mutation with host molecular immune response remains unknown. Therefore, we sought to investigate the transcriptome response of COVID-19 patients infected with SARS-CoV-2 KR-mutant (KR-Patients) and wild-type SARS-CoV-2 (RG-Patients) compared to healthy controls without infection (Additional file 2: Table S1 and Additional file 1: Fig. S1). As reported [13], the KR-patients displayed significantly higher viral load and more severe cases (defined by ICU admission) than RG-patients (Fig. 1A, B). By using RNA extracted from nasopharyngeal swabs, more than 30 million reads were generated for each sample from KR-patients, RG-patients, and Healthy controls by transcriptome sequencing. Among them, more than 85% of reads mapped to the human genome were thus used for transcriptome profiling.

The differential gene expression analysis (adjusted *p*-value (*q*-value < 0.05) and log2-fold change (log2 FC > 1.5)) was then performed comparing KR-patients with healthy controls (KR versus Healthy KR-v-H) RG-patients with healthy controls (RG versus Healthy RG-v-H), and KR-patients with RG-patients (KR versus RG KR-v-RG) (see Additional file 2: Tables S2-4 for all significant differentially expressed (DE) genes). The principal component analysis (PCA) revealed that compared to RG-patients, most KR-patients tended to form a separate cluster (Fig. 1C), indicating the different attributes of DE genes in KR-patients. The distance clustering based on the expression level of cytokine and interferon-stimulated (ISG) genes, the majority of the KR-patients grouped in a cluster while the RG-patient and healthy control samples were more scattered (Fig. 1D). We identified a markedly higher number of DE genes (DE genes = 2082) in KR-patients (KR-vs-H) than that in RG-patients (DE genes = 370, RG-vs-H), suggesting that KR-mutant SARS-CoV-2 infection profoundly perturbed transcriptome of COVID-19 patients (Fig. 1E and Additional file 2: Table S2-4). Noteworthy, the COVID-19 patient cohorts used in this study are from the first wave of the pandemic before vaccination and hence free from any interference due to vaccine response. By direct comparison of KR-patients with RG-patients (KR versus RG), we also found a higher number of up-regulated DE genes (up = 2338) that overlapped with DE genes from comparison of KR-patients with healthy controls (Additional file 1: Fig. S2A, B). We found a robust overexpression of numerous genes including interferons, cytokine, and immune-related genes in KR-patients (Fig. 1F).

### Pathway analysis reveals hyper-immune response in KR-patients

**Fig. 2:**
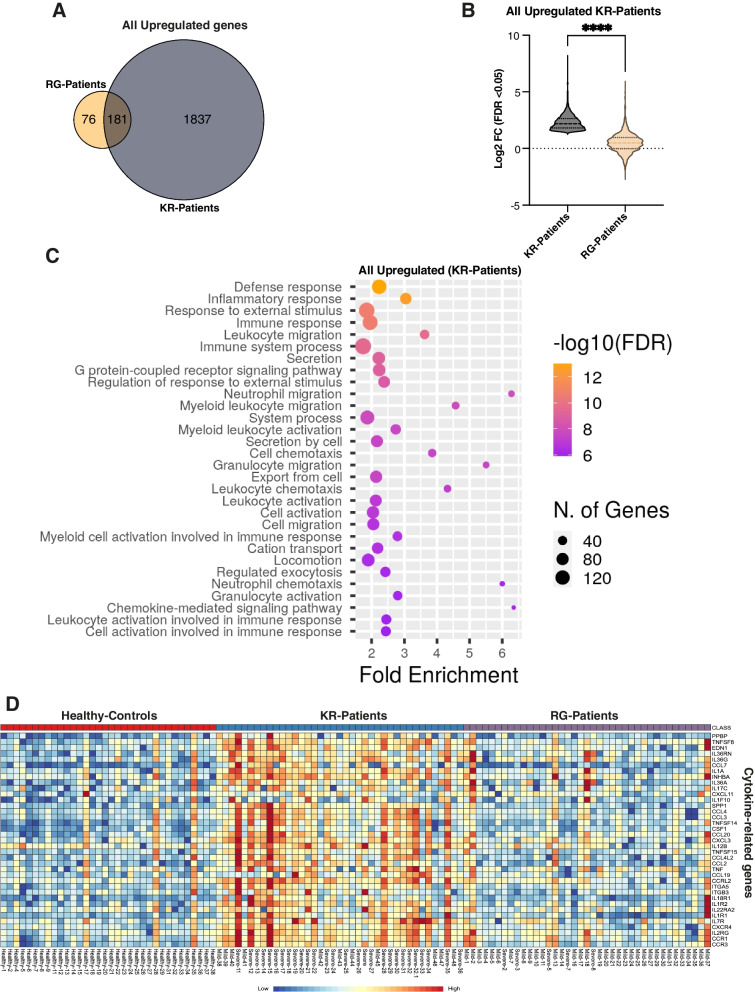
Hyper-expression of immune response genes including cytokines in KR-Patients.** A** Venn diagram shows the number of all up-regulated genes (adj *p*-value < 0.05) between KR-patients and RG-patients. **B** Violin plot shows log2 fold-change values of all significantly (adj *p*-value < 0.05) up-regulated genes in KR-patients. DE genes are highly up-regulated in KR-patients compared to RG-patients. *p*-values were calculated using unpaired two-tailed *t*-test, ****P =  < 0.0001. **C** Dot plot showing GO-enrichment analysis (top 30 GO-Biological Processes BP enriched pathways are shown) for significantly up-regulated genes in KR-patients (adj *p*-value < 0.05 and log2 fold-change ≥ 2). The FDR is based on nominal *p*-value from the hypergeometric test. Fold Enrichment corresponds to the percentage of genes in the pathway list, divided by the corresponding percentage in the background. GO term analysis was performed by ShinyGO (version 0.074). **D** Heatmap of significantly differentially expressed cytokine-related genes. KR-Patients (*n* = 39), RG-Patients (*n* = 39), and Healthy controls (*n* = 34). The sample names show COVID-19 disease status (Severe = ICU/Deceased, Mild = Not admitted to ICU, and Healthy)

Next, we compared all significantly up-regulated genes in KR-patients and RG-patients (Fig. 2A). The majority of up-regulated genes (181 out of 257) in RG-patients (RG-vs-H) overlapped with the gene set from KR-patients (KR-vs-H). After co-normalization between samples, up-regulated genes showed significantly higher log_2_ fold change values in KR-patients than in RG-patients (Fig. 2B).

We then performed gene ontology (GO) and functional pathway enrichment analysis on all significantly up-regulated genes in the KR-patients using ShinyGO [38]. A graphical representation of enriched biological process pathways for significantly up-regulated genes in the KR-patients group is shown in Fig. 2C. Pathway enrichment analysis showed an overrepresentation of biological processes associated with immune responses (Fig. 2C, Additional file 1: Fig. S2C, and Additional file 2: Table S5). Indeed, the most enriched biological process pathways (based on FDR) are defense response, inflammatory response, response to external stimulus, immune response, immune system process, and different immune cell activation and migration (Fig. 2C). Furthermore, we performed a comparative gene ontology (GO) enrichment analysis using all significant DE genes (up- and down-regulated) in KR-patients (KR-vs-H), RG-patients (RG-vs-H), and KR versus RG patients. We observed that all enriched biological processes related to inflammatory response and cytokines production/secretion were strongly up-regulated in KR-patients compared to the RG-patients group (Additional file 1: Fig S2C and Fig. S3), indicating a highly robust immune response in COVID-19 patients infected with KR-mutant SARS-CoV-2.

By comparing the significant DE genes of KR-patients nasopharyngeal transcriptome with previously reported bronchoalveolar fluid (BALF) transcriptome from severe COVID-19 patients [16], we found 149 common DE genes (Additional file 1: Fig. S4A). Pathway enrichment analysis of these common DE genes (Additional file 1: Fig. S4B) exhibited overrepresentation of biological processes related to chemokine signaling, immune response, and other cytokine mediated responses (Additional file 1: Fig. S4B).

### Cytokines and interferon-stimulated genes (ISGs) display heightened expression in KR-patients

To understand the difference in cytokine profile between the KR-patients and the RG-patients, we listed significant differentially expressed cytokine-related genes in both conditions. Expression levels of multiple cytokine-related genes were more significantly up-regulated in the KR-patients than in the RG-patients groups (Fig. 2D). Among these up-regulated cytokine-related genes in KR-patients, we predominantly found chemokines (CCL7, PPBP, CXCL11, CCL2, CCL3, CCL4, CCL20, CXCL3, CCL4L2, and CCL19), chemokine receptors (CCRL2, CXCR4, CCR1, and CCR3), interleukin (IL36G, IL1A, IL36A, IL17C, IL1F10, and IL12B), interleukin receptors (IL36RN, IL18R1, IL1R1, IL1R2, IL22RA2, IL7R, and IL2RG), tumor necrosis factor (TNFSF8, TNFSF14, TNFSF15, and TNF), and colony-stimulating factor (CSF1) (Fig. 2D). Some of these cytokine-related genes were also previously reported in patients with severe COVID-19 [16, 39, 40] (Additional file 1: Fig. S4C). Furthermore, we observed high-level up-regulation of CCL2 and CCL7 chemokines in KR-patients, which play a critical role in monocyte recruitment [41] and have shown to be linked with higher viral load [16].

We then compared interferon (IFN) responses between KR-patients and RG-patients. By overlapping significantly up-regulated genes in the KR-patients with a previously reported list of 628 ISGs [42], we found 44 ISGs in the up-regulated KR-patients list (Additional file 1: Fig. S4D). Notably, the majority of these ISGs belong to a previously recognized [42] cluster of inflammation regulators (Additional file 1: Fig. S4E), which is consistent with a report of highly expressed ISGs in BALF samples from severe COVID-19 patients [16] (Additional file 1: Fig. S4F). We observed significantly elevated expression of these ISGs in KR-patients than in RG-patients (Additional file 1: Fig. S4G), indicating a potent IFN response. This robust IFN response of inflammation-related ISGs may be linked to immunopathogenesis in the KR-patients.

### KR-mutant alone is capable of enhancing the expression of immune response genes

**Fig. 3:**
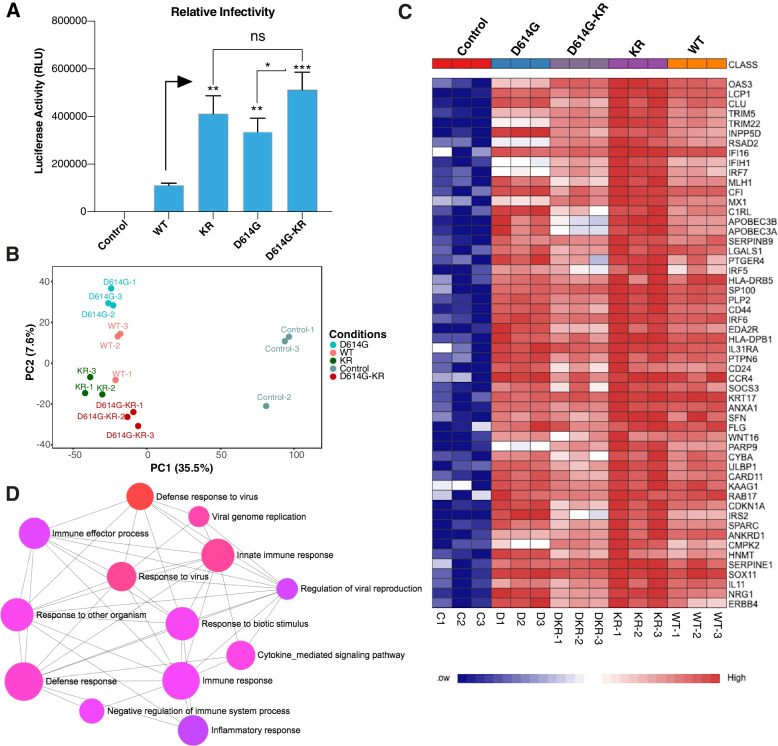
Transcriptome analysis of cells incubated with SARS-CoV-2 virus-like particles (VLP).** A** Bar chart shows relative infectivity as measured by luciferase detection assay (± SD from *n* = 4 independent experiments, [unpaired two-sided *t*-test, *p*-values KR-vs-WT:0.0025 (**), D614G-vs-WT:0.0024 (**), D614G-KR-vs-WT:0.0007 (***), and non-significant between KR and D614G-KR (ns)]). **B** PCA on transcriptome of HEK293T cell lines (expressing ACE2-TMPRSS2) incubated with four variants (WT, KR, D614G, and D614G-KR) SARS-CoV-2 virus-like particles and control (not incubated). PCA plot is based on normalized all differentially expressed (DE) genes and autoscaling of data was used (*n* = 3 independent experiments). **C** Heatmap shows normalized expression of top significant differentially expressed genes (comparing KR versus control) in KR, WT, D614G, D614G-KR VLP incubated, and control HEK293T cell lines (adj *p*-value < 0.05 and log2 fold-change cutoff ≥ 1.5) (*n* = 3 independent experiments). **D** GO-enrichment analysis (top 13 biological processes pathways based on *p*-value and FDR are shown) of up-regulated genes in KR SARS-CoV-2 VLP incubated cells. The enriched terms display an interconnected network with overlapping gene sets (from the list). Each node represents an enriched term and colored by its *p*-value from red to blue in ascending order (red shows the smallest *p*-value). The size of each node corresponds to number of linked genes from the list

To understand whether KR-mutation is directly linked with the observed hyper-immune response, we utilized a hybrid alphavirus-SARS-CoV-2 (Ha-CoV-2) virus-like particle (VLP) approach [34]. We performed comparative analysis using wild-type (WT) VLP containing reference sequences of all four structural proteins (S, M, N, and E) and KR-mutant (KR) VLP with two mutations R203K/G204R in the nucleocapsid protein. Two additional controls, D614G-KR (containing both R203K/G204R and spike D614G mutations) and D614G (only spike D614G mutation), were also included (Additional file 1: Fig. S5). The spike mutation D614G was used as a control because it was shown to be linked with high infectivity and viral load [43, 44]. These VLPs were incubated with HEK293T cell lines (stably expressing ACE2 and TMPRSS2) to test their infectivity through the expression level of a luciferase reporter gene (Additional file 1: Fig. S5 and Fig. 3A). We observed that KR VLP alone or combined with D614G (D614G-KR) displayed enhanced infectivity compared to WT VLP (Fig. 3A).

By transcriptome analysis of these VLPs incubated cells, we identified 884, 802, 668, and 739 significant differentially expressed (DE) genes (adjusted *p*-value < 0.05 and log2 fold-change ≥ 1.5) in the KR, D614G, D614G-KR, and WT SARS-CoV-2 VLP incubated cells, respectively, (Additional file 2: Table S6-9). The transcriptome profiles of SARS-CoV-2 VLPs incubated cells display a distinct pattern from the control (not infected cells) (Fig. 3B). Like the KR-patient samples, we found a robust overexpression of defense and immune response-related genes, including cytokines and ISGs, in the KR VLP incubated cells (Fig. 3C–D) that overlapped with DE genes from KR-patients (Additional file 1: Fig. S6A). By comparing all significant DE genes between KR, D614G, D614G-KR, and WT, we found 44 common DE genes between KR, D614G-KR, and WT conditions (Additional file 1: Fig. S6B). The majority of these showed high-level up-regulation in KR VLP incubated cells (Additional file 1: Fig. S6C). Pathway enrichment analysis of the up-regulated genes in KR (Additional file 1: Fig. S6C) exhibited an overrepresentation of biological processes such as interferon signaling, defense response, and response to the virus (Additional file 1: Fig. S6D and Additional file 2: Table S10). These results suggest that KR mutant is associated with enhanced expression of immune and inflammatory response genes.

We also tested the impact on host cell transcriptome by only overexpressing the N gene (with and without 203K/204R mutations). We identified 83 and 67 differentially expressed genes (adjusted *p*-value < 0.05 and fold-change cutoff ≥ 1) in the N-KR mutant and N-wild-type transfected HEK293T cells, respectively (Additional file 1: Fig. S7A-B). Consistent with our previous report [13], we observed a high-level expression of immune response and interferon-related genes in the N-KR transfected cells (Additional file 1: Fig. S7C). Most of these up-regulated genes overlapped with KR VLP transcriptome data (Additional file 1: Fig. S7D). Similar to KR VLP cells, all up-regulated genes in the N-KR transfected cells were enriched in pathways related to antiviral immune response (Additional file 1: Fig. S7E).

### Proteomic profiling shows enhanced expression of immune response proteins in KR-VLP incubated cells

**Fig. 4:**
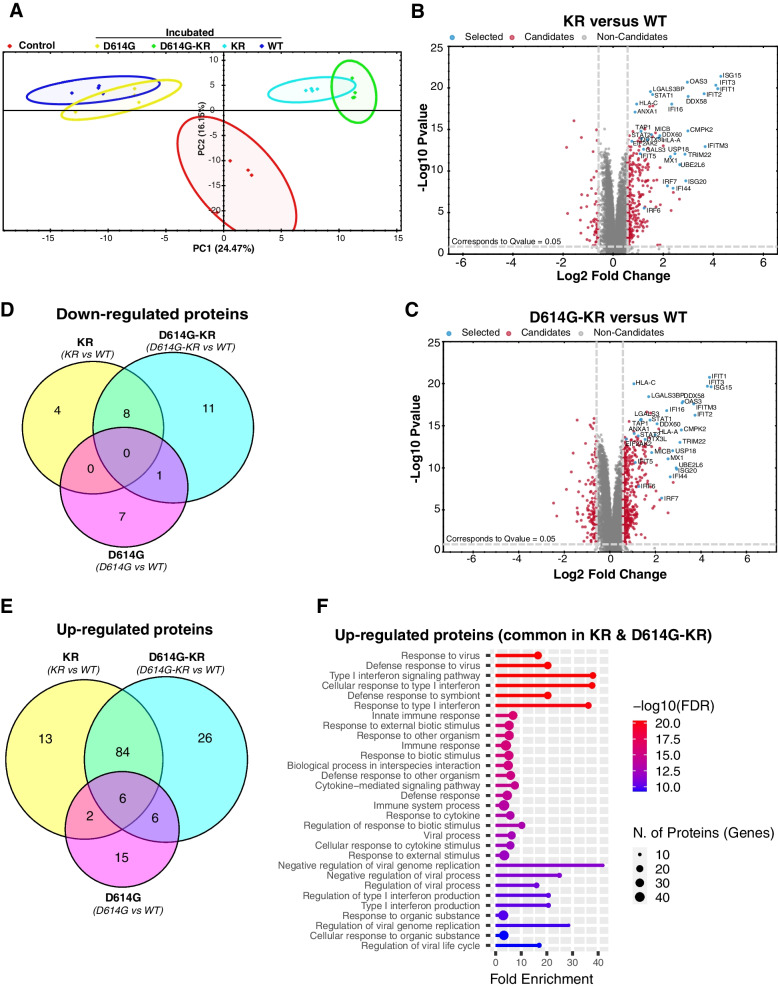
KR-incubated cells display enhanced expression of interferon-stimulated and immune processes related proteins. **A** PCA on proteomic data (obtained by data independent acquisition (DIA) mass spectrometry) of control HEK293T cell lines and incubated with four SARS-CoV-2 VLP variants (WT, KR, D614G, and D614G-KR). PCA plot is generated by Spectronaut software version 15) and is based on all differentially expressed (DE) proteins (*n* = 4 independent experiments). **B–C** Volcano plot displaying differentially expressed proteins. **B** Comparing KR VLP incubated cells with WT (KR versus WT). **C** Comparing D614G-KR VLP incubated cells with WT (D614G-KR versus WT). Proteins with statistically significant (adjusted *p*-value ≤ 0.05) differences between KR/D614G-KR mutant and WT conditions are shown in red (candidates) and all other non-significant proteins (non-candidates) are shown in gray. The names of top differentially expressed proteins common between KR and D614G-KR conditions are shown and highlighted in blue (selected). **D** Venn diagram shows the overlap of all significantly (adj *p*-value < 0.05 and Log2 ratio ≥ 1) down-regulated proteins between KR, D614G-KR, and D614G SARS-CoV-2 VLP incubated cells. **E** Venn diagram shows the overlap of all significantly (adj p-value < 0.05 and Log2 ratio ≥ 1) up-regulated proteins between KR, D614G-KR, and D614G SARS-CoV-2 VLP-infected cells. **F** Plot showing GO-enrichment analysis (top 30 GO-Biological Processes (BP) enriched pathways are shown) for significantly up-regulated proteins common between KR and D614G-KR conditions (adj *p*-value < 0.05 and log2 fold-change ≥ 1). GO term analysis was performed by ShinyGO (version 0.074)

We next investigated the impact of KR at the protein level by comparative proteomic profiling of SARS-CoV-2 VLP incubated cells containing KR, D614G, D614G-KR mutations, and WT (Wuhan genotype). We identified around 6000 total proteins across all replicates (*n* = 4) in all samples (Additional file 1: Fig. S8). Principal component analysis showed that biological replicates clustered together in all samples, and VLP incubated samples were clearly separated from the control (not treated) samples (Fig. 4A). Notably, the KR and D614G-KR incubated samples clustered more closely than WT and D614G samples (Fig. 4A), indicating similarity between KR and D614G-KR based on common KR mutations. The proteome changes in KR, D614G-KR, and D614G were quantified by comparing them with cells incubated with WT VLP (see Additional file 2: Table S11 for all differentially expressed proteins in all conditions). The KR and D614G-KR samples displayed a comparable pattern of proteome changes (Fig. 4B, C). The common up-regulated proteins related to immune response are highlighted in Fig. 4B, C. We catalogued significantly (adj *p*-value < 0.05 and log2 ratio >  = 1) up-regulated (KR = 106, D614G-KR = 124, and D614G = 29) and down-regulated proteins (KR = 12, D614G-KR = 21, and D614G = 8) (Fig. 4D, E and Additional file 2: Table S11).

Among the group of significantly up-regulated proteins, 84 are shared between KR and D614G-KR samples (Fig. 4E). Similar to transcriptomic data, the majority of these significantly up-regulated proteins (Fig. 4E) include most typical ISGs such as ISG15/20, IFIT1/2/3/5/M3, IFI16/44, OAS3, and MX1, as well as interferon regulated transcription factors STAT1/2, IRF6, and IRF7 (Fig. 4E and Additional file 1: Fig. S9). Gene ontology enrichment analysis of the common significantly up-regulated proteins revealed highly enriched biological processes (bp) related to immune response (Fig. 4F and Additional file 2: Table S12). The top enriched terms include “response to virus,” “defense response,” “type 1 interferon signaling”, “cellular response to type 1 interferon”, and “innate immune response” (Fig. 4F). All enriched immune response-related pathways clustered together, indicating many shared proteins (Additional file 1: Fig. S10). Further, GO term analysis of the shared genes between KR transcriptome and proteome data (Additional file 1: Fig. S11A) revealed activation of interconnected biological processes that are predominantly enriched in innate immune response, defense response, interferon signaling, and cytokine response (Additional file 1: Fig. S11B). By comparing the enriched (GO terms) pathways from KR transcriptome and KR proteome, we observed that majority of the KR transcriptome pathways overlapped with proteome data (Additional file 1: Fig. S11C).

### KR mutation leads to enhance cytokines response in VLP incubated cells

Next, we investigated and compared the level of proinflammatory cytokines between KR-VLP and WT-VLP incubated cell lysates. We found that KR-VLP cells display significantly higher concentrations of proinflammatory cytokines (IFN Gamma, IL-8, IL-6, and IL-1) than control and the WT-VLP incubated cells (Additional file 1: Fig. S12). These findings further confirmed the activation of similar pathways and cellular events at both the gene and protein levels upon incubation with KR-mutant SARS-CoV-2 VLP.

### KR-patients display elevated neutrophil-to-lymphocyte ratio (NLR) predicted by transcriptome data

**Fig. 5:**
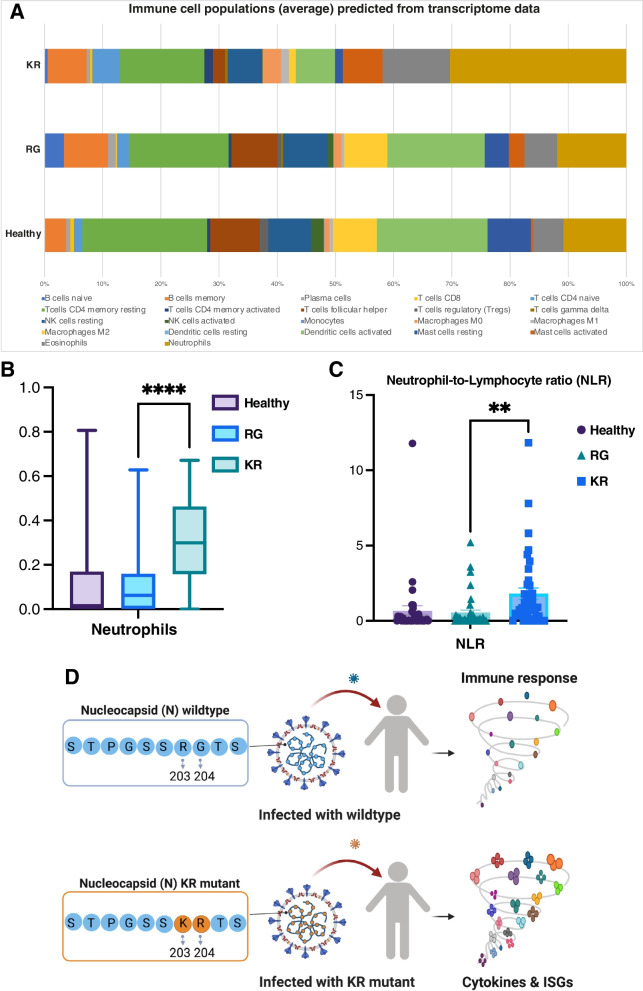
Immune cell composition predicted from transcriptome data and schematic summary.** A** The average proportion of immune cell types predicted from transcriptome data. **B** The proportion of neutrophils predicted from transcriptome data. Asterisks show significant difference (****p-values < 0.0001, paired two-sided *t*-test KR-vs-RG). **C** The neutrophil to lymphocyte ratio (NLR) predicted from transcriptome data. The proportion of lymphocyte corresponds to the sum of proportions of T cells, B cells, and NK cells. The *p*-values (** < 0.0062), calculated by paired two-sided *t*-test, show significant difference between KR and RG samples. **D** Schematic representation summarizing the main findings. The KR mutant SARS-CoV-2 infected patients display high magnitude of immune response

Given the enhanced level of cytokines in both KR-patients and KR-VLP incubated cells, we sought to estimate the composition of immune cells in the patient’s nasopharyngeal transcriptome data by CIBERSORT (a computational pipeline able to predict and quantify immune cell composition from bulk transcriptomic data) [33]. Using transcriptome data, we predicted the proportion of different immune cell types in healthy controls, RG-patients, and KR-patients (Fig. 5A and Additional file 1: Fig. S13). We found that KR-patients showed significantly higher levels of neutrophils than the RG-patients (Fig. 5B and Additional file 1: Fig. S13). Recent reports highlighted the association of neutrophil-to-lymphocyte (NLR) ratio with COVID-19 disease severity and mortality [45, 46]. In our analysis, we observed notably higher NLR in KR-patients than RG-patients (Fig. 5C and Additional file 1: Fig. S13), further indicating the link of KR-mutation with hyper-inflammatory responses.

These findings are consistent with the reported higher neutrophils and NLR in BALF samples from severe COVID-19 patients [16], thus supporting the usefulness of our nasopharyngeal transcriptome data in assessing the host immune response and cell composition.

## Discussion

Despite the considerable increase in genomic monitoring of global COVID-19 cases, it remains challenging to link new SARS-CoV-2 mutations with its pathogenesis and other clinical implications due to the scarcity of COVID-19 patient metadata in public databases. Recently, we found that two consecutive amino acid substitutions (R203K/G204R) in the nucleocapsid (N) protein are linked with increased viral load and severity in COVID-19 patients in Saudi Arabia during the early wave of the pandemic [13]. The current study performed a comparative transcriptomic analysis of COVID-19 patients infected with KR-mutant (KR-patients) and wild-type SARS-CoV-2 (RG-patients). We observed enhanced expression of immune response genes in KR-patients. By further experimental characterization in VLP incubated cells, we verified the link of KR mutation with enhanced inflammatory responses.

The molecular mechanism underlying the ability of the KR mutation to contribute to exacerbated host immune responses remains unclear. The KR mutation is present within a conserved serine–arginine (SR) rich linker region (LKR). This SR domain is critical for phosphorylation-dependent regulation of N protein functional activities during SARS-CoV-2 infection cycle [47, 48]. The changes of the original “RGT” to the “KRT” motif and the specific enhancement of phosphorylation at the adjacent serine 206 site [13] in the mutant N protein might play a role. Overexpression of only the N gene KR mutant in cell culture was able to enhance the expression level of interferons and other immune response genes. The high dose of N protein has been shown to promote type I interferon (IFN-I) and inflammatory cytokines expression [49]. In this context, the reported higher viral load [13] and higher infectivity [22] of KR mutant could result in an increased quantity of phosphorylated N protein that may be linked with heightened immune responses. The KR substitutions within N could be linked with enhanced replication or transcription, which may result in higher viral load and hyper-inflammatory responses. Notably, few of the RG patients display similar profiles to KR patients, which could be due to underlying severe disease conditions. Moreover, the increased interaction of N KR-mutant with different host proteins that are associated with signaling pathways and viral processes might be involved [13]. Another study reported NLRP3 inflammasome activation and induction of hyper-inflammatory response mediated by N protein [50]. The potential link of N protein KR mutations with inflammasome activation needs further investigation. Recently, SARS-CoV-2 N protein was shown to bind cell surface and modulates innate and adaptive immune responses. The ability of SARS-CoV-2 nucleocapsid protein to transfer to neighboring non-infected cell surfaces [51] could potentially augment KR mutant activity.

While multiple reports have provided an ample understanding of the immune transcriptomics in COVID-19 patients [52–59], little is known about the potential differences in host immune responses to variant forms of SARS-CoV-2 due to the lack of variant information in those studies. Transcriptomic studies reported dissimilar immune phenotypes of COVID-19 patient cohorts. Some indicated impaired interferon (IFN) response with decreased IFN-α/β expression [60–62] while others showed enhanced type I IFN and pro-inflammatory reactions in severe COVID-19 patients [16, 63, 64]. Among other factors, distinct viral genotypes could also contribute to the discrepancies in immune responses of COVID-19 patient cohorts. The host immune response in viral infection is like a two-edged sword; on the one hand, the healthy immune response is essential for fighting pathogens but heightened responses can cause detrimental outcomes [65] such as respiratory failure, tissue injury, and death due to excessive discharge of pro-inflammatory cytokines [39, 66, 67] known as “cytokine storm”. We found significantly elevated levels of pro-inflammatory cytokines and hyper-expression of various interferon-stimulated and cytokine genes in KR mutant background (Fig. 5D). However, the non-availability of blood leukocytes or BALF samples is the main limitation because the nasopharyngeal swabs transcriptome may not precisely represent the complete immune response.

## Conclusions

Together, the higher NLR and hyper-expression of proinflammatory cytokines and ISGs in KR patients possibly explain the connection of cytokine storms with observed disease severity and excessive deaths (in this patient cohorts) during the early peak of the pandemic in the absence of herd immunity. The identified link of N protein KR mutation with heightened host inflammatory and cytokine responses may contribute to understanding SARS-CoV-2 pathogenesis and developing efficient therapeutics and vaccines with broader protection against new emerging SARS-CoV-2 variants and other future human coronaviruses.

## Supplementary Information

**Additional file 1: Fig. S1.** Information of the patient cohorts and healthy controls used for metatranscriptome analysis. **Fig. S2.** Comparative transcriptome analysis of KR-Patients and RG-Patients. **Fig. S3.** Significant pathways identified for DE genes of KR-patients and RG-patients. **Fig. S4.** Comparison of COVID-19 patient nasopharyngeal transcriptome with BALF transcriptome data. **Fig. S5.** Schematic of Ha-CoV-2 VLPs and experiment design. **Fig. S6.** Transcriptome profiling of cells incubated with SARS-CoV-2 virus-like particles (VLP). **Fig. S7.** Transcriptome analysis of N-KR mutant and N-wildtype HEK293T transfected cells. **Fig. S8.** Data independent acquisition (DIA) mass spectrometry from VLP incubated cells. **Fig. S9.** Selected interferon and immune processes related proteins identified by proteomic analysis in VLP incubated cells. **Fig. S10.** Hierarchical clustering tree of enriched pathways. **Fig. S11.** Comparison of transcriptome and proteome of VLP incubated cells. **Fig. S12.** KR-mutation increases cytokines in VLP incubated cells. **Fig. S13.** Immune cell populations predicted from transcriptome data.

**Additional file 2: Table S1.** Demorgraphic and clinical information of all samples. **Table S2.** All significant DE genes (KR-patients versus Healthy). **Table S3.** All significant DE genes (RG-patients versus Healthy). **Table S4.** All DE genes (KR-patients versus RG-patients ). **Table S5.** Transcriptome Pathways enrichment (biological processes). **Table S6.** Significant DE genes KR-VLP versus control. **Table S7.** Significant DE genes D614G-VLP versus control. **Table S8.** Significant DE genes D614G-KR-VLP versus control. **Table S9.** Significant DE genes WT-VLP versus control. **Table S10.** Pathways enrichment (biological processes). **Table S11.** All DE proteins in KR, D614G-KR, and D614G versus WT. **Table S12.** Proteome Pathways enrichment (biological processes).

## Data Availability

The raw sequencing data including nasopharyngeal transcriptomes from patients and RNA-seq of VLP incubated HEK-293 T cells have been uploaded to European Nucleotide Archive (https://www.ebi.ac.uk/ena/) under the bioproject accession number PRJEB57869 at the URL https://www.ebi.ac.uk/ena/browser/view/PRJEB57869 [68]. RNA-seq data from HEK-293 T cells transfected with plasmids overexpressing nucleocapsid proteins are available under accession number PRJEB44716 URL: https://www.ncbi.nlm.nih.gov/bioproject/?term=PRJEB44716 [69]. Sequences of assembled virus genomes are publicly available at GISAID (Additional file 2: Table S1). The mass spectrometry proteomics data have been deposited to the ProteomeXchange Consortium via the PRIDE partner repository with the dataset identifier PXD034557 at the URL https://www.ebi.ac.uk/pride/archive/projects/PXD034557 [70].

## Abbreviations

COVID-19: Coronavirus disease 2019
SARS-CoV-2: Severe acute respiratory syndrome coronavirus 2
RNA-seq: RNA sequencing
VLP: Virus-like particle
GISAID: Global Initiative on Sharing Avian Influenza Data
ISG: Interferon-stimulated gene
IFN-I: Type I interferon
SR-LKR: Serine–arginine (SR) rich linker region (LKR)
NLR: Neutrophil-to-lymphocyte ratio
IFN Gamma: Interferon gamma
IL-8: Interleukin 8
IL-1: Interleukin 1
IL-6: Interleukin 6
ICU: Intensive care unit
GO terms: Gene Ontology terms
Ha-CoV-2: Hybrid alphavirus-SARS-CoV-2 virus-like particle
BALF: Bronchoalveolar fluid
