# Supplementary material for: Impact of the SARS-CoV-2 nucleocapsid 203K/204R mutations on the inflammatory immune response in COVID-19 severity
Source: Genome Med. 2023 Jul 21;15:54. doi: 10.1186/s13073-023-01208-0 (PMC10360309; doi:10.1186/s13073-023-01208-0)
Supplement: Supplementary file 1 — Additional file 1: Fig. S1. Information of the patient cohorts and healthy controls used for metatranscriptome analysis. Fig. S2. Comparative transcriptome analysis of KR-Patients and RG-Patients. Fig. S3. Significant pathways identified for DE genes of KR-patients and RG-patients. Fig. S4. Comparison of COVID-19 patient nasopharyngeal transcriptome with BALF transcriptome data. Fig. S5. Schematic of Ha-CoV-2 VLPs and experiment design. Fig. S6. Transcriptome profiling of cells incubated with SARS-CoV-2 virus-like particles (VLP). Fig. S7. Transcriptome analysis of N-KR mutant and N-wildtype HEK293T transfected cells. Fig. S8. Data independent acquisition (DIA) mass spectrometry from VLP incubated cells. Fig. S9. Selected interferon and immune processes related proteins identified by proteomic analysis in VLP incubated cells. Fig. S10. Hierarchical clustering tree of enriched pathways. Fig. S11. Comparison of transcriptome and proteome of VLP incubated cells. Fig. S12. KR-mutation increases cytokines in VLP incubated cells. Fig. S13. Immune cell populations predicted from transcriptome data. [file 13073_2023_1208_MOESM1_ESM.pdf]

Supplementary Information

Additional file 1: Supplementary Materials for: Impact of the SARS-CoV-2 nucleocapsid 203K/204R mutations on the inflammatory immune response in COVID-19 severity.

This file includes Supplementary Figures: Fig. S1-S12

Fig. S1. Information of the patient cohorts and healthy controls used for metatranscriptome analysis.
Fig. S2. Comparative transcriptome analysis of KR-Patients and RG-Patients.
Fig. S3. Significant pathways identified for DE genes of KR-patients and RG-patients.
Fig. S4. Hyper-expression of ISGs in KR-patients.
Fig. S5. Transcriptome profiling of cells incubated with SARS-CoV-2 virus-like particles (VLP).
Fig. S6. Data independent acquisition (DIA) mass spectrometry from VLP incubated cells.
Fig. S7. Selected interferon and immune processes related proteins identified by proteomic analysis in VLP incubated cells.
Fig. S8. Hierarchical clustering tree of enriched pathways.
Fig. S9. Comparison of transcriptome and proteome of VLP incubated cells.
Fig. S10. KR-mutation increases cytokines in VLP incubated cells.
Fig. S11. Immune cell populations predicted from transcriptome data.

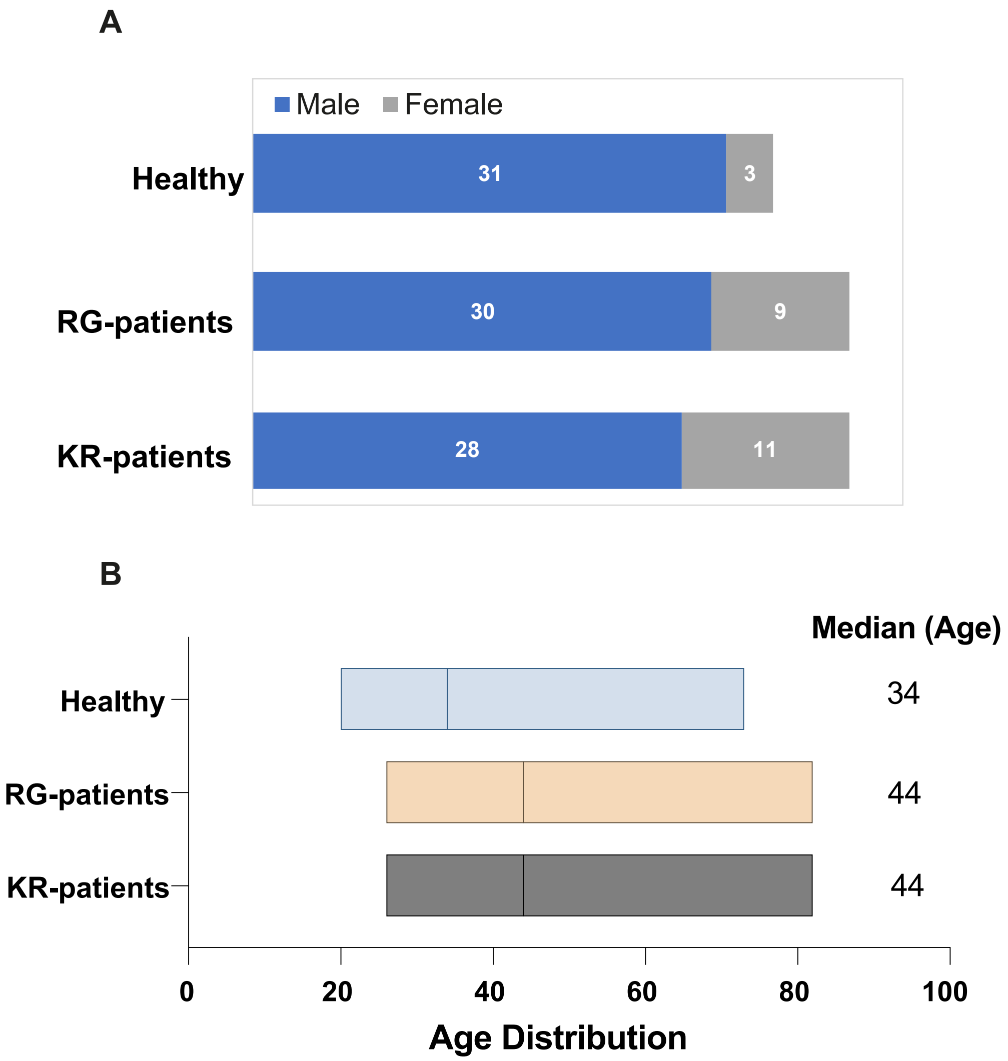

Fig. S1. Information of the patient cohorts and healthy controls used for metatranscriptome analysis. A COVID-19 patients infected with SARS-CoV-2 KR-mutant having 203K/204R mutations in nucleocapsid (KR-Patients n = 39) and SARS-CoV-2 wildtype having 203R/204G reference amino acids (RG-Patients n = 39) and healthy controls (Healthy n = 34). **B** Age distribution of sample cohorts.

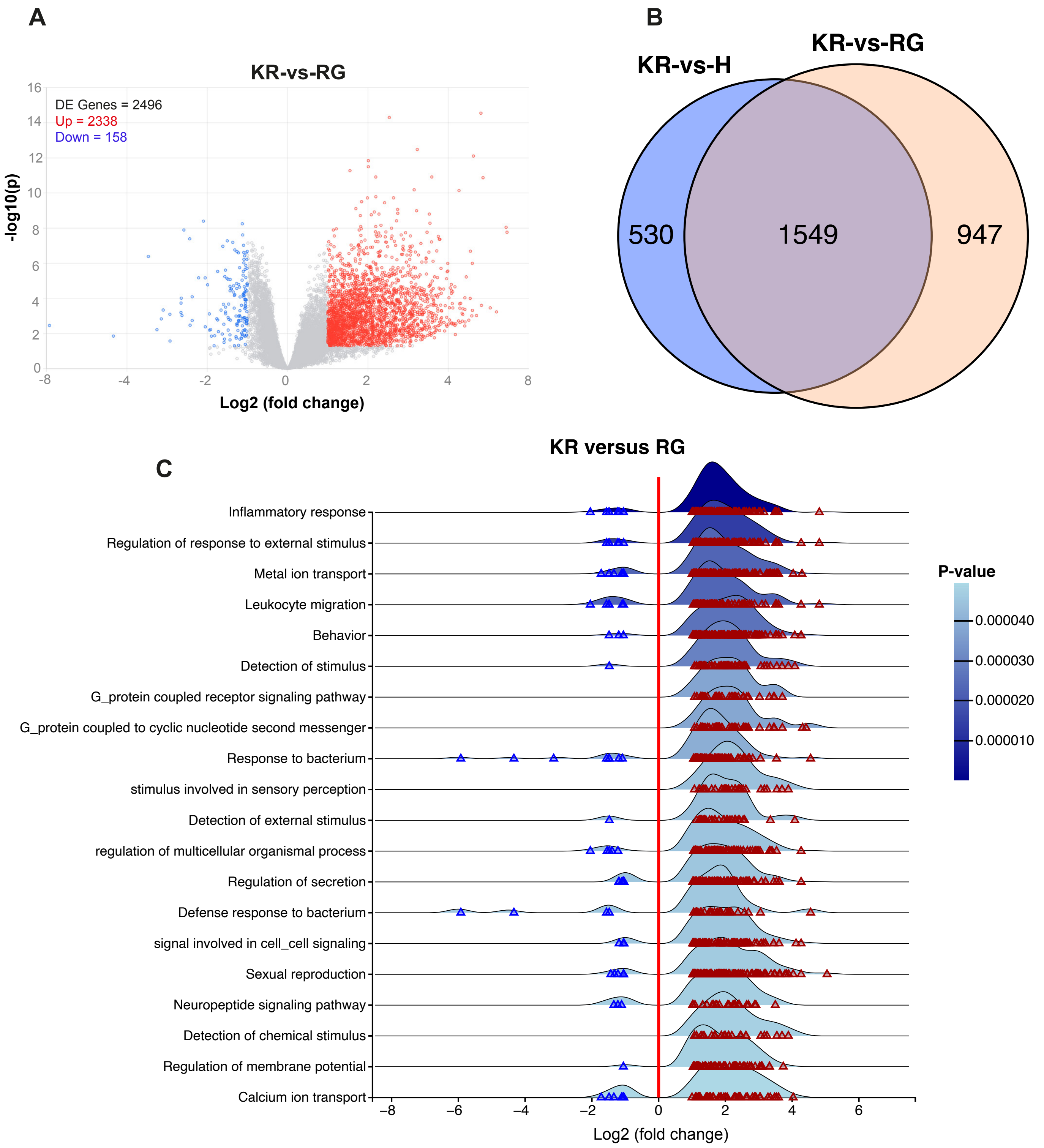

Fig. S2. Comparative transcriptome analysis of KR-Patients and RG-Patients. A Volcano-plot showing significant (adj p-value < 0.05 and log2 fold-change cutoff ≥ 1.5) differentially expressed (DE) genes comparing KR-Patients versus RG-Patients (KR-vs-RG). Genes with significant up-regulation are shown in red and down-regulated are shown in blue. **B** Venn diagram shows the number of DE genes (adj p-value <0.05) between KR-vs-H and KR-vs-RG comparison. **C** Ridgeline plot showing the distribution of log2_foldchange values for all DE genes (KR-vs-RG) and significantly enriched pathways. The distribution for each pathway is colored according to the pathway's adjusted P-value. The vertical triangles show DE genes, up-regulated genes are in red and downregulated genes are shown in blue.

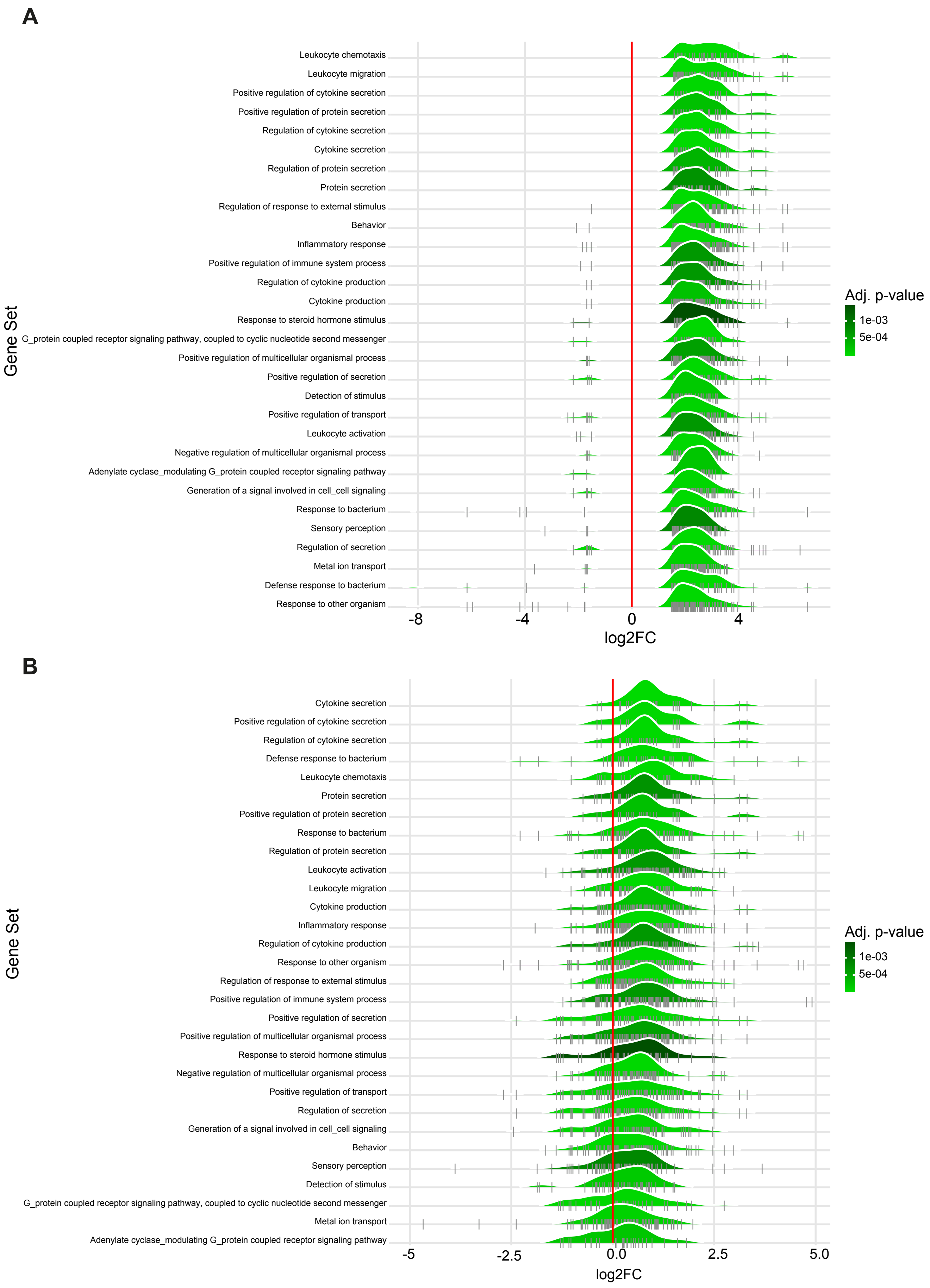

Fig. S3. **Significant pathways identified for DE genes of KR-patients and RG-patients.** Ridgeline plot showing the distribution of log2_foldchange values for all DE genes **A** (KR-patients versus Healthy) and **B** (RG-patients versus Healthy) in the significantly enriched pathways. The distribution for each pathway is colored according to the pathway's adjusted P-value. The vertical gray lines indicate the log2_foldchange values of all DE genes in the enriched pathways.

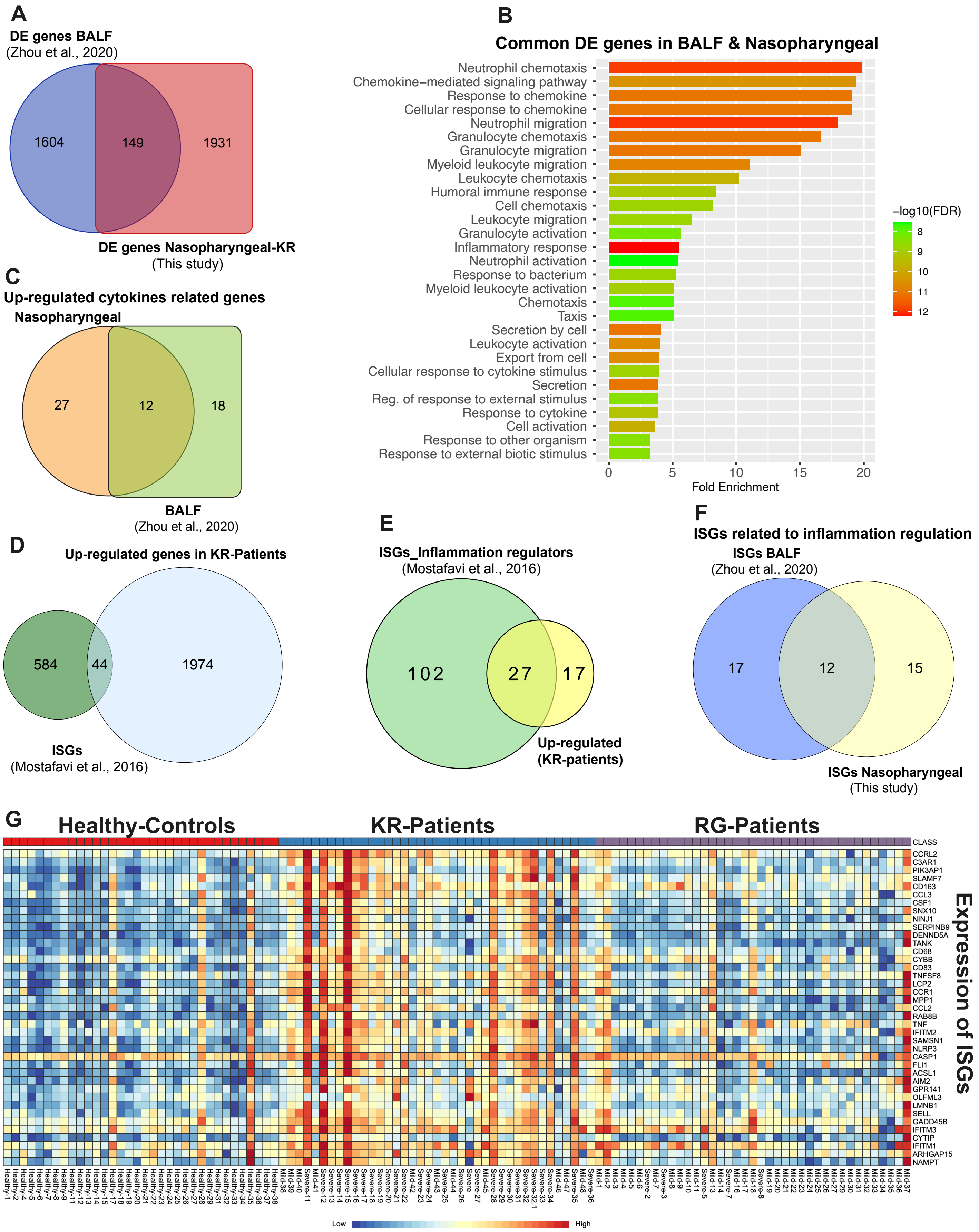

Fig. S4. Comparison of COVID-19 patient nasopharyngeal transcriptome with BALF transcriptome data. A Venn diagram shows the overlap of all DE genes in KR-patients transcriptome with DE genes from BALF transcriptome reported in Zhou et al., 2020 (16). **B** Plot showing GO-enrichment analysis (top 30 GO-Biological Processes (BP) enriched pathways are shown) for common DE genes between KR-patients transcriptome and BALF transcriptome. GO term analysis was performed by ShinyGO (version 0.061). **C** Venn diagram of all significantly (adj p-value <0.05) up-regulated cytokine related genes in KR-patients nasopharyngeal transcriptome with up-regulated cytokines in BALF data (Zhou et al., 2020) (16). **D** Venn diagram shows the overlap of all significantly (adj p-value <0.05) up-regulated genes in KR-patients with reported list composed of 628 ISGs (Mostafavi et al., 2016). **E** Venn diagram shows the overlap of ISGs cluster related to inflammation regulation (Mostafavi et al., 2016) and up-regulated genes in KR-patients. **F** Venn diagram shows the overlap of ISGs related to inflammation regulation between KR-patients nasopharyngeal and BALF transcriptome data (Zhou et al., 2020) (16). **G** Heatmap of highly upregulated ISGs in KR-Patients comparing to Healthy controls.

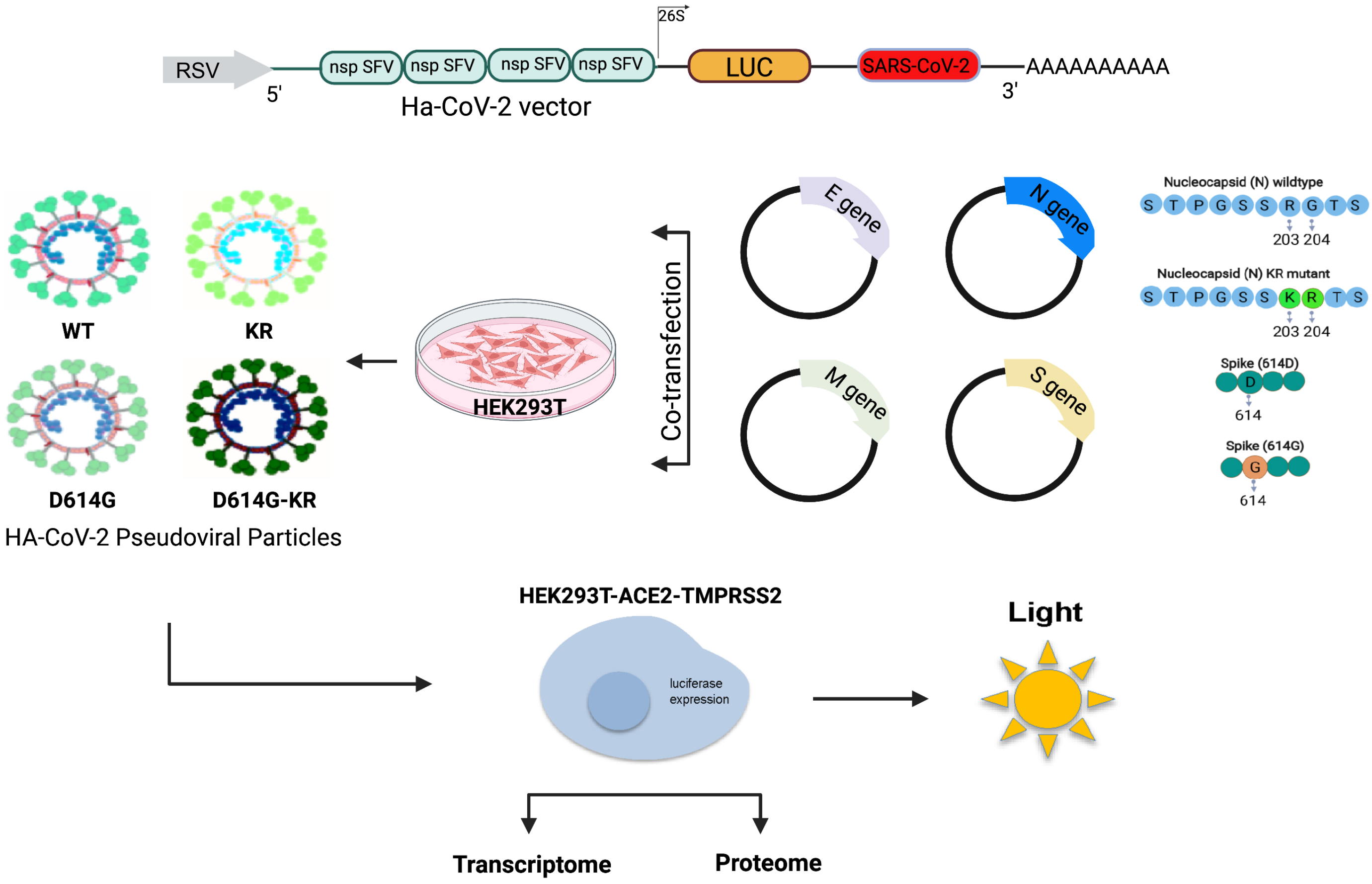

Fig. S5. Schematic of Ha-CoV-2 VLPs and experiment design. The schematic Illustration of Ha-CoV-2 construct was adapted from Hetrick et al., 2022 (34). The RSV (Rous Sarcoma Virus) promoter transcribes the complete viral RNA genome for packaging. In the diagram, 5′ untranslated, Semliki Forest virus (SFV) nonstructural proteins (nsps1-4) open-reading frames, promoter for Luc reporter, SARS-CoV-2 packaging sequence, 3′ untranslated region, and a poly(A) tail having self-cleavage site for the hepatitis delta virus ribozyme (RZ) are shown. Co-transfection of Ha-CoV-2 and plasmid expressing four SARS-CoV-2 structural proteins (N, E, M, and S) into HEK293T produce pseudoviral particles. The four different custom-made (Virongy) hybrid alphavirus-SARS-CoV-2 (Ha-CoV-2) virus-like particles (VLP) are shown. Ha-CoV-2-WT (WT) contains wildtype sequences of all 4 structural proteins (S, M, N, and E). Ha-CoV-2-KR (KR) having nucleocapsid protein with two mutations R203K/G204R. Ha-CoV-2-D614G (D614G) having mutation in spike and Ha-CoV-2-D614G-KR contains both mutant KR nucleocapsid protein and spike D614G mutation. Workflow showing incubation of ACE2-TMPRSS2-expressing HEK293T cells with above mentioned four virus-like particles (VLPs).

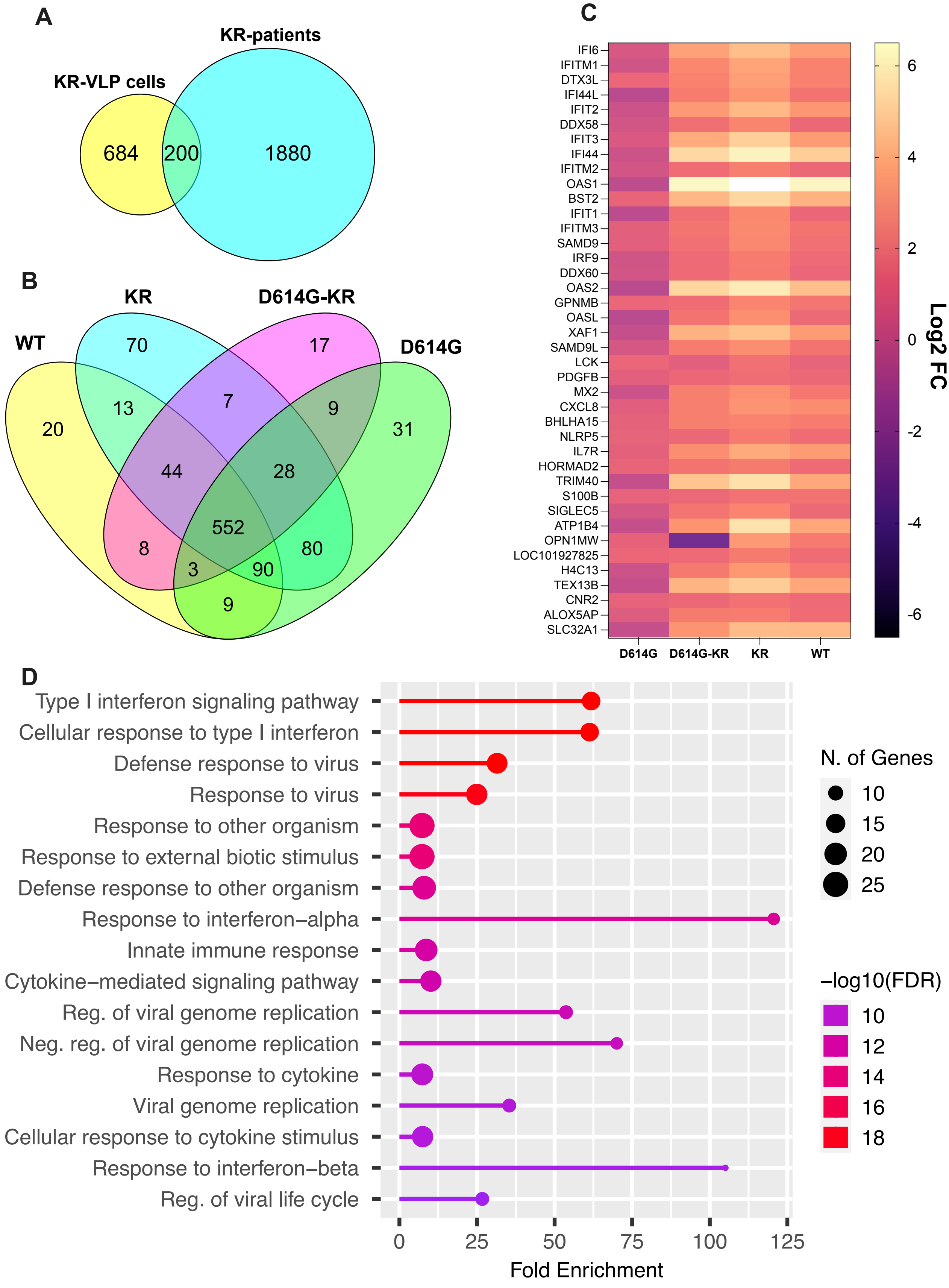

Fig. S6. Transcriptome profiling of cells incubated with SARS-CoV-2 virus-like particles (VLP). **A** Venn diagram shows the overlap of all DE genes (adj p-value <0.05 and Log2 foldchange >= 1.5) in KR-patients transcriptome and KR-mutant SARS-CoV-2 VLP treated cells. **B** Venn diagram shows the number of all DE genes (adj p-value <0.05 and Log2 foldchange >= 1.5) in KR, D614G-KR, and D614G SARS-CoV-2 VLP infected cells. **C** Heatmap shows log2 foldchange of up-regulated genes common between KR, D614G-KR, and WT conditions. **D** Plot showing enriched pathways for genes shown in (C).

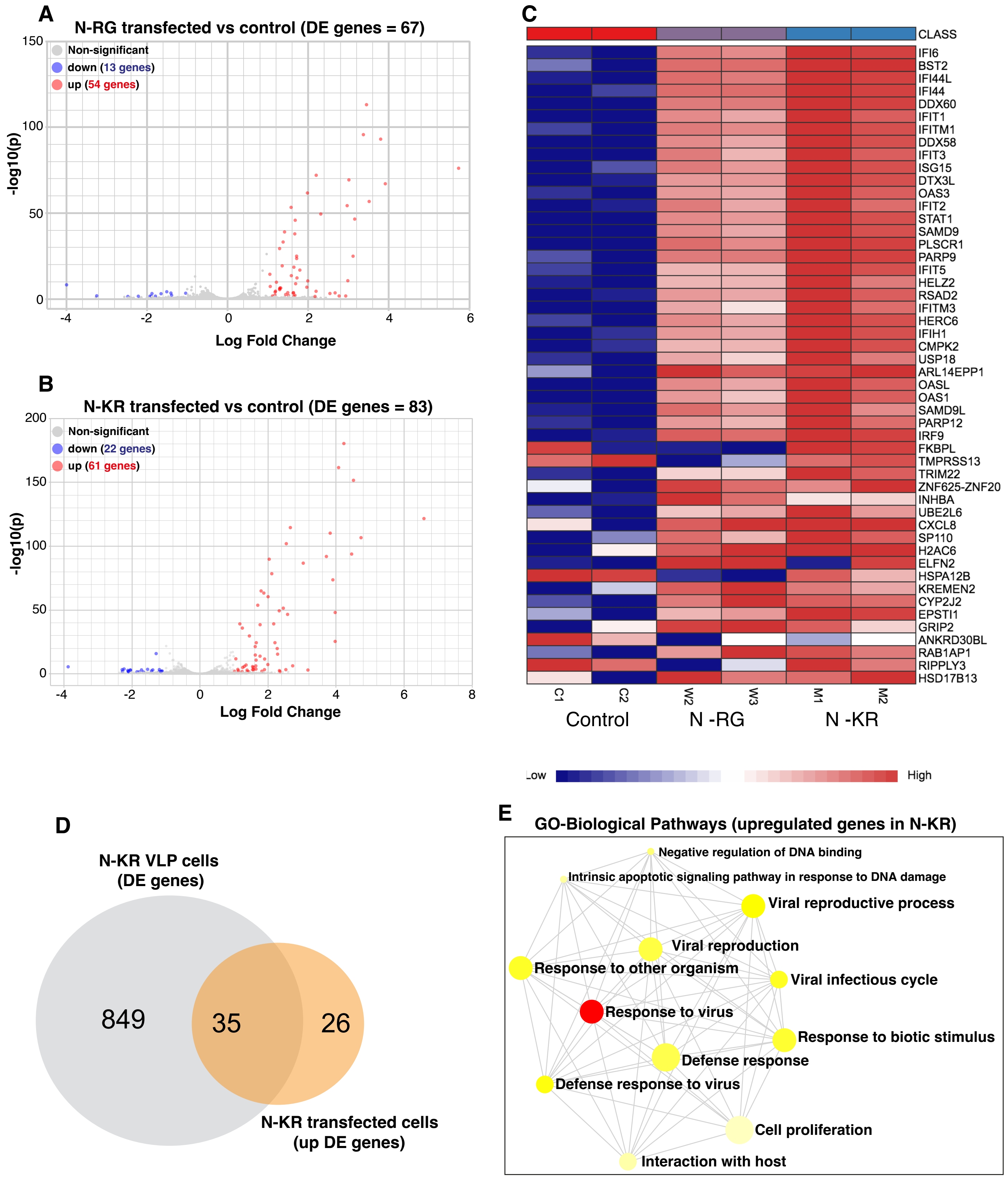

Fig. S7. Transcriptome analysis of N-KR mutant and N-wildtype HEK293T transfected cells. HEK293T cells were transfected with plasmids expressing the full-length N-wildtype and N-KR mutant gene plus mock control for 48 hours. **A, B** Volcano-plot showing differentially expressed DE (adj p-value < 0.05 and fold-change cutoff ≥ 1). Up-regulated genes are shown in red and down-regulated are shown in blue. **C** Heatmap showing expression level of top DE genes (N-KR mutant versus control). **D** Venn diagram shows the overlap of N-KR transfected cells up-regulated genes with KR VLP DE genes. **E** GO enrichment analysis of up-regulated genes in the N-KR mutant condition. The enriched terms display an interconnected network with overlapping gene sets. Each node represents an enriched term, colored by its p-value (red shows smallest p-value) and size corresponds to number of genes.

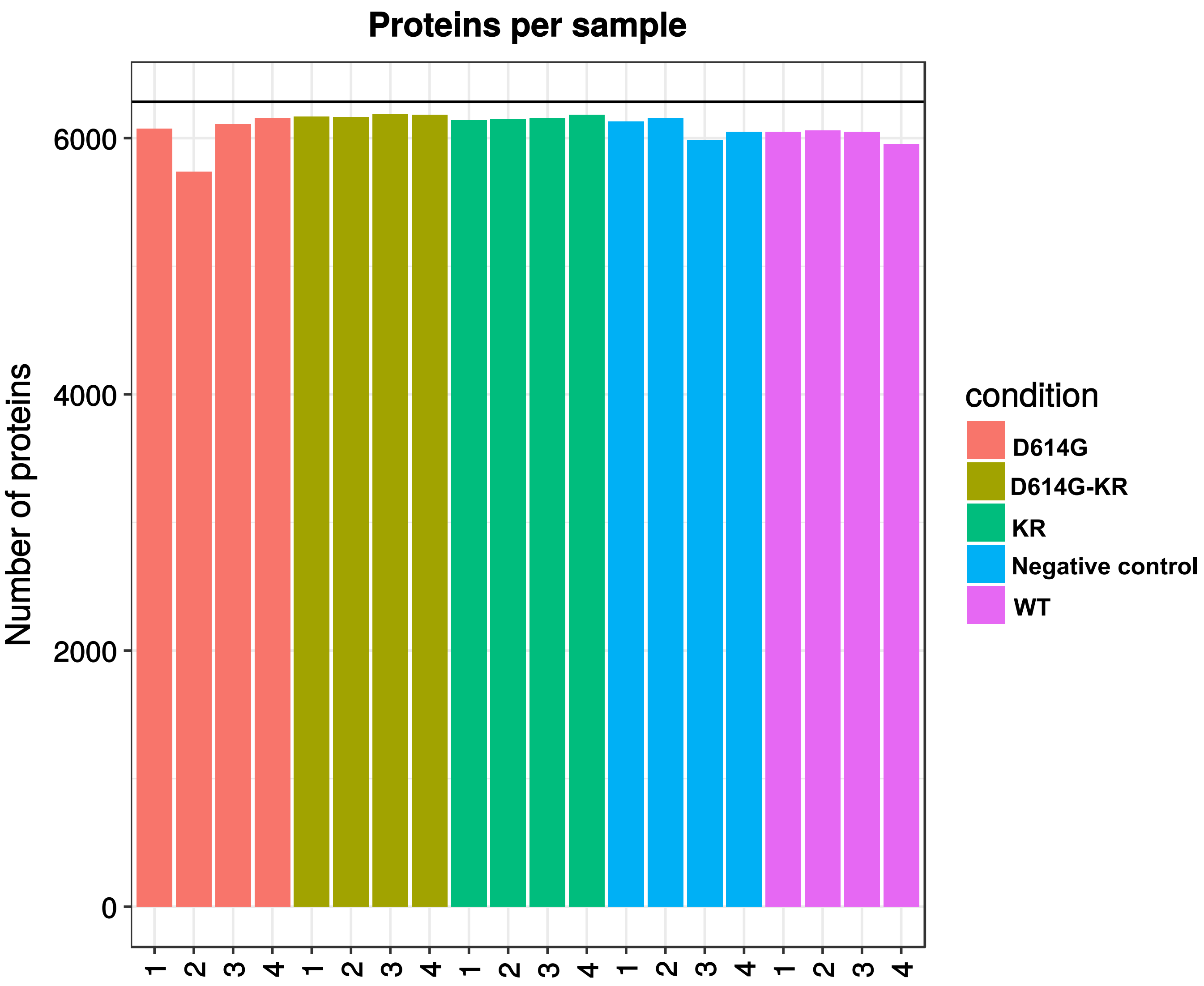

Fig. S8. Data independent acquisition (DIA) mass spectrometry from VLP incubated cells. Plot shows number of proteins identified in all conditions (Control, WT, KR, D614G, and D614G-KR).

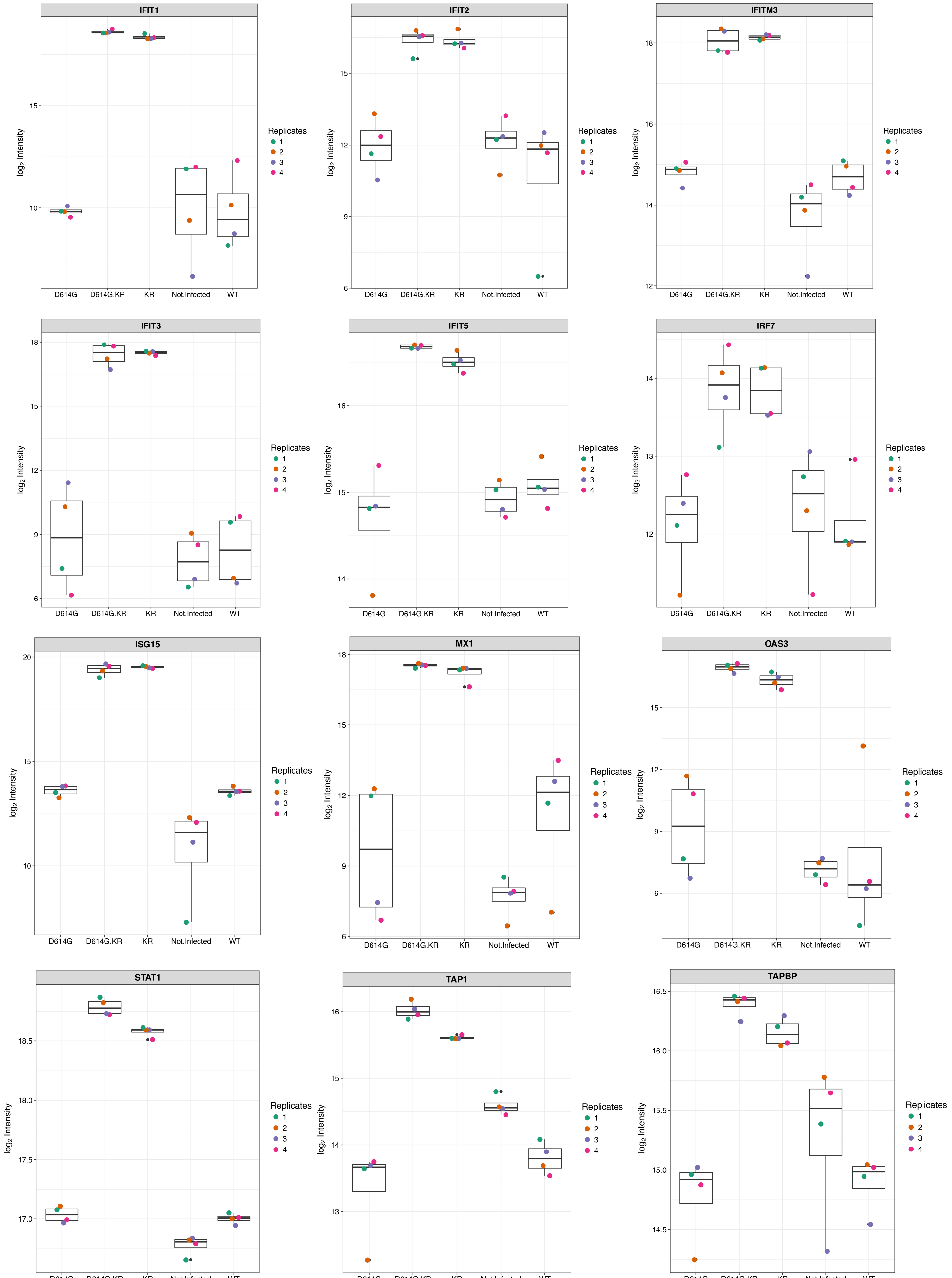

Fig. S9. Selected interferon and immune processes related proteins identified by proteomic analysis in VLP incubated cells.

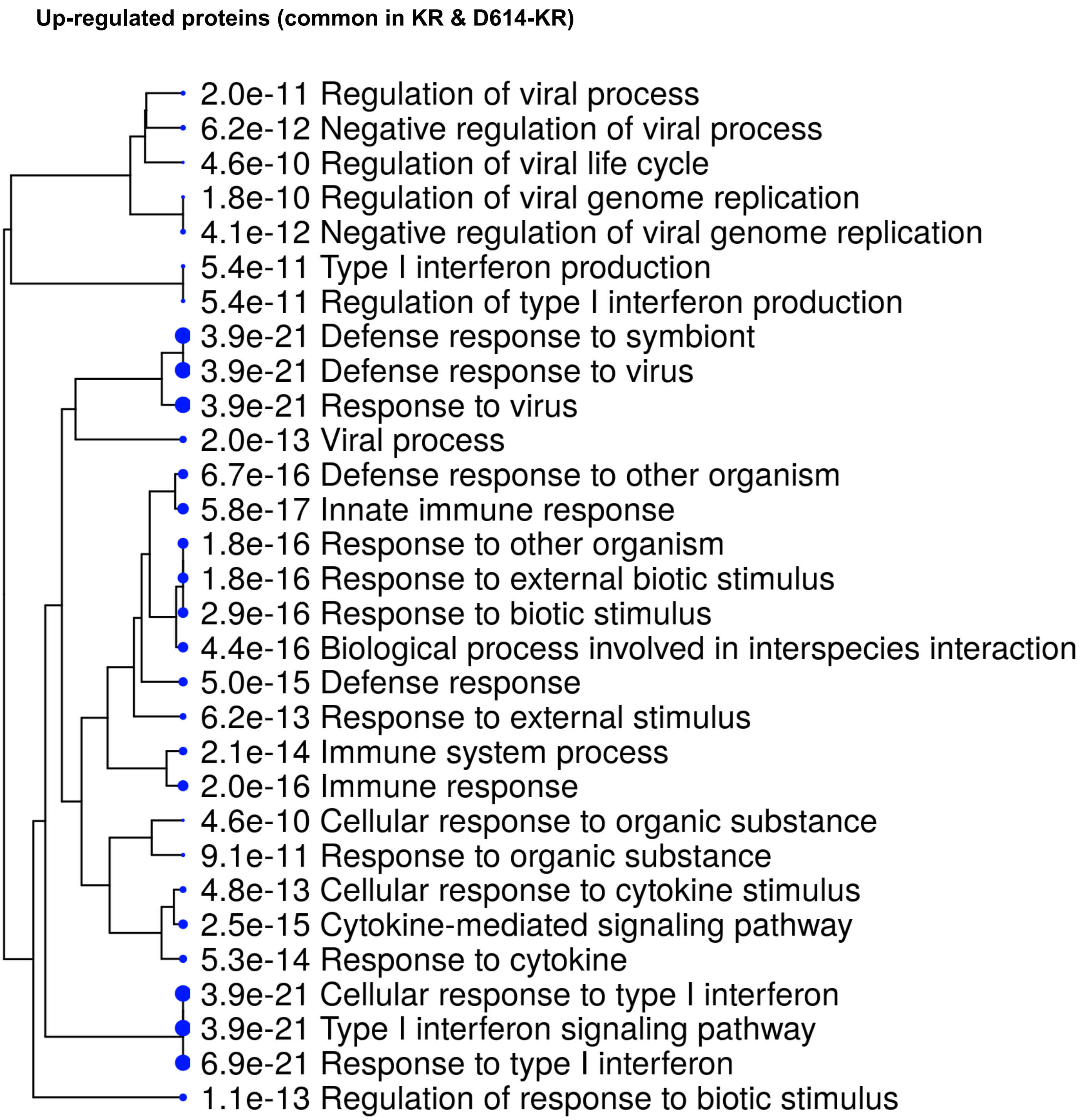

Fig. S10. Hierarchical clustering tree of enriched pathways. The clustering tree summarizing the correlation among significant pathways listed in Figure 4F. Pathways with many shared proteins are clustered together. Bigger dots indicate more significant P-values.

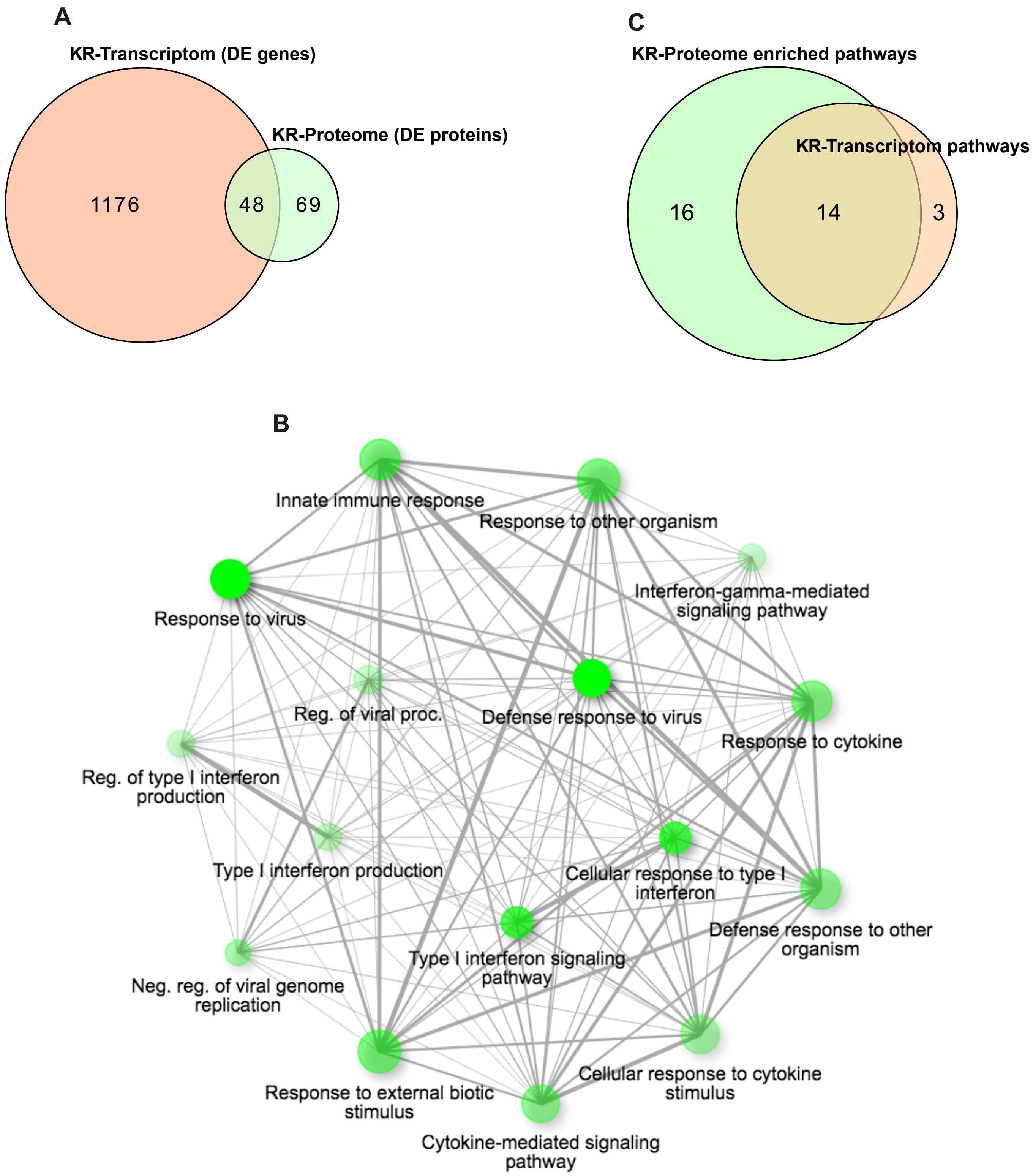

Fig. S11. Comparison of transcriptome and proteome of VLP incubated cells. A Venn diagram shows the overlap of transcriptome and proteome data of KR-mutant SARS-CoV-2 VLP infected cells. **B** Network plot showing enriched pathways for common genes between transcriptome and proteome data of KR-mutant SARS-CoV-2 VLP infected cells as shown above. Related pathways (nodes) are inter-connected, darker nodes represent more significantly enriched gene sets, size of node shows number gene sets, and thicker edges indicate more overlapped genes. **C** Venn diagram shows the overlap of transcriptome and proteome enriched pathways from KR VLP infected cells.

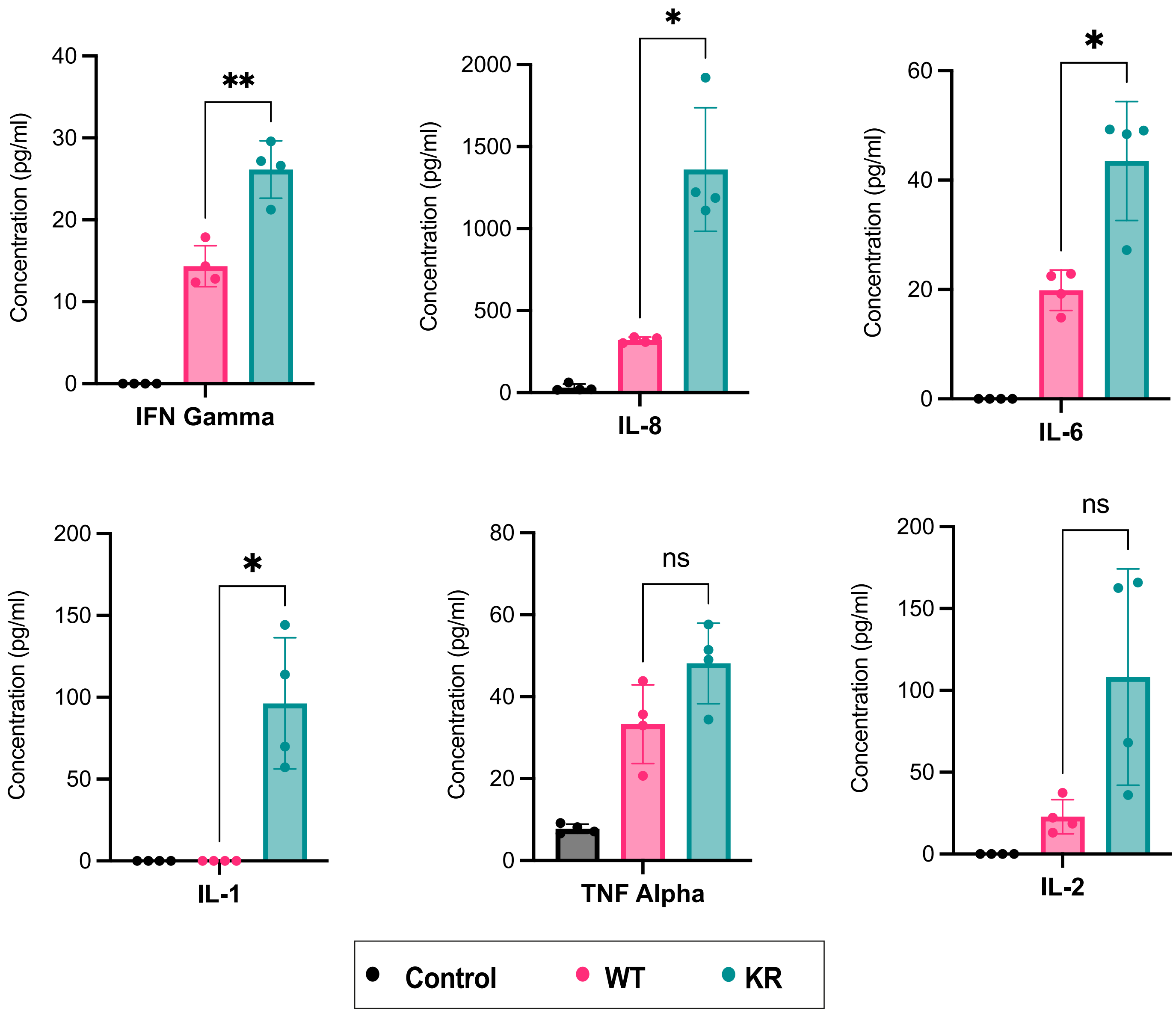

Fig. S12. KR-mutation increases cytokines in VLP incubated cells. Concentration of cytokines (IFN Gamma, IL-8, IL-6, IL-1, TNF-alpha, and IL-2) in the cell lysates of control, KR, and WT VLP infected cells (n =4) determined by flowcytometry using multiplex cytokine storm kit (AssayGenie, HUAMCOV05). Asterisks show significant difference between KR and WT VLP (**p-values = 0.0085 IFN Gamma, *p-values = 0.0129 IL-8, *p-values = 0.0385 IL-6, and *p-values = 0.0172 IL-1) as determined by two-sided t-test.

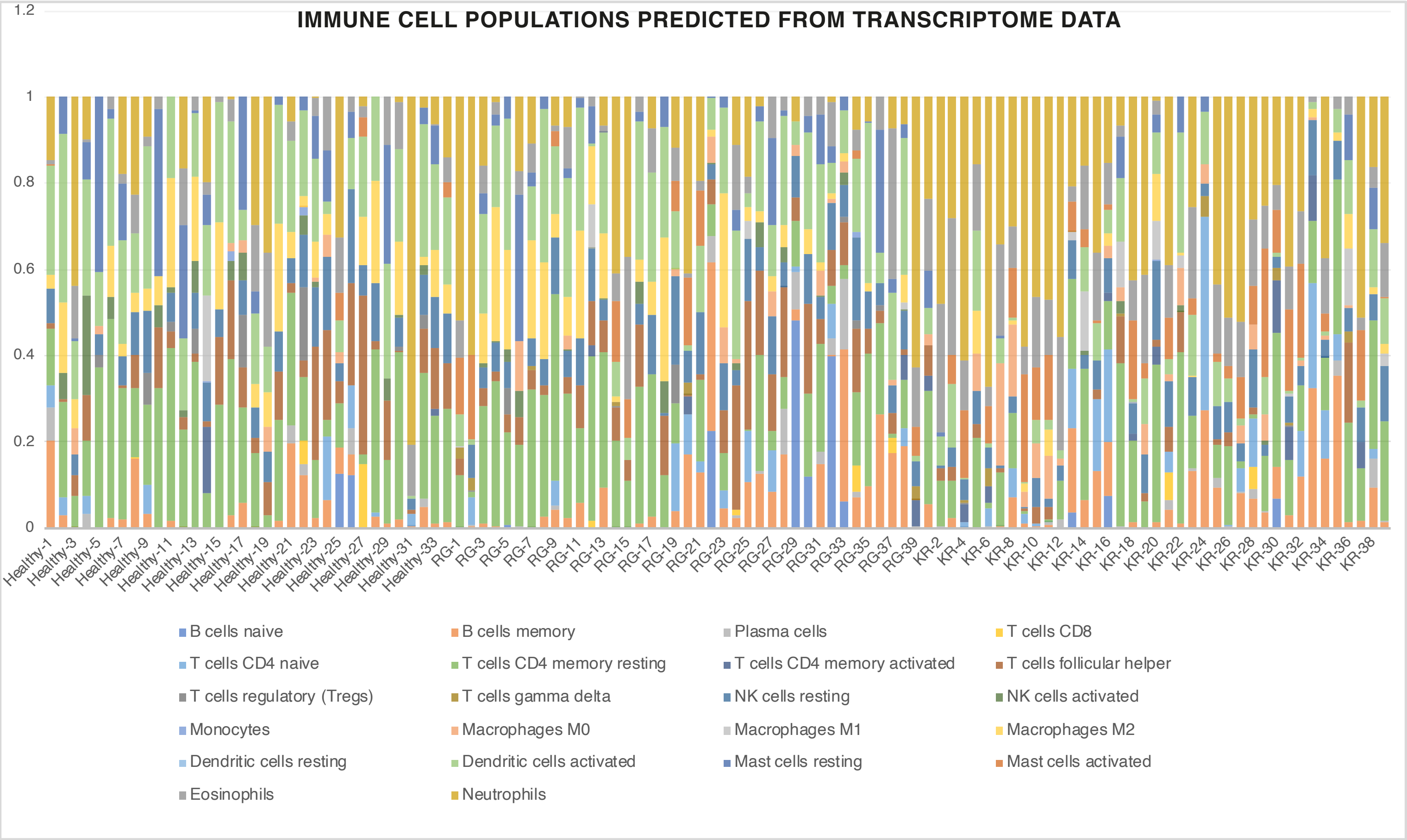

Fig. S13. Immune cell populations predicted from transcriptome data. The proportion of immune cell types in all individual samples predicted from transcriptome data by CIBERSORT (Newman et al., 2015).
